# Model-based virtual patient analysis of human liver regeneration predicts critical perioperative factors controlling the dynamic mode of response to resection

**DOI:** 10.1186/s12918-019-0678-y

**Published:** 2019-01-16

**Authors:** Babita K. Verma, Pushpavanam Subramaniam, Rajanikanth Vadigepalli

**Affiliations:** 1 Daniel Baugh Institute for Functional Genomics/Computational Biology, Department of Pathology, Anatomy, and Cell Biology, Thomas Jefferson University, Philadelphia, PA USA; 2 Department of Chemical Engineering, Indian Institute of Technology-Madras, Chennai, India

**Keywords:** Liver regeneration, Dynamic modeling, Level of resection, Metabolic load, Cell death sensitivity, Phase portrait

## Abstract

**Background:**

Liver has the unique ability to regenerate following injury, with a wide range of variability of the regenerative response across individuals. Existing computational models of the liver regeneration are largely tuned based on rodent data and hence it is not clear how well these models capture the dynamics of human liver regeneration. Recent availability of human liver volumetry time series data has enabled new opportunities to tune the computational models for human-relevant time scales, and to predict factors that can significantly alter the dynamics of liver regeneration following a resection.

**Methods:**

We utilized a mathematical model that integrates signaling mechanisms and cellular functional state transitions. We tuned the model parameters to match the time scale of human liver regeneration using an *elastic net* based regularization approach for identifying optimal parameter values. We initially examined the effect of each parameter individually on the response mode (normal, suppressed, failure) and extent of recovery to identify critical parameters. We employed phase plane analysis to compute the threshold of resection. We mapped the distribution of the response modes and threshold of resection in a virtual patient cohort generated in silico via simultaneous variations in two most critical parameters.

**Results:**

Analysis of the responses to resection with individual parameter variations showed that the response mode and extent of recovery following resection were most sensitive to variations in two perioperative factors, metabolic load and cell death post partial hepatectomy. Phase plane analysis identified two steady states corresponding to recovery and failure, with a threshold of resection separating the two basins of attraction. The size of the basin of attraction for the recovery mode varied as a function of metabolic load and cell death sensitivity, leading to a change in the multiplicity of the system in response to changes in these two parameters.

**Conclusions:**

Our results suggest that the response mode and threshold of failure are critically dependent on the metabolic load and cell death sensitivity parameters that are likely to be patient-specific. Interventions that modulate these critical perioperative factors may be helpful to drive the liver regenerative response process towards a complete recovery mode.

**Electronic supplementary material:**

The online version of this article (10.1186/s12918-019-0678-y) contains supplementary material, which is available to authorized users.

## Background

Liver has the ability to fully regenerate post liver injury or surgical resection [1]. This process of regeneration takes place via a unique mechanism in which differentiated hepatocytes re-enter the cell cycle to replenish lost cells [2]. This is followed by proliferation of non-parenchymal cells to eventually reconstitute the cell types in the liver tissue, and tissue remodeling to re-establish the lobular scale morphology [3]. This unique regenerative capacity enables majority of clinical interventions into liver disease via surgical resection, as well as live donor transplants and small-for-size liver transplants. While liver regeneration is robust in healthy individuals, chronic disease diminishes this regenerative capacity making the surgical interventions less than effective and can even lead to post-surgery liver failure in some patients [4, 5]. There is an unmet need for a prognostic tool that takes into account the patient-specific, disease-modified, regenerative capacity and evaluates these in the context of known activation of molecular pathways and cellular events post resection. A limited set of studies focused on developing a mathematical model of the underlying cellular and molecular mechanisms as a potential solution to this problem [6–14]. These studies demonstrate that a unified set of cellular and molecular mechanisms, with associated parameters, can account for differences in the regenerative response to varying levels of resection [15], with failure above a threshold [16].

Majority of the computational modeling studies have focused on the relatively short time scale of regenerative response in the rat and mouse (hours to few days), and hence are not correctly tuned to account for the weeks-to-months time scale relevant to the human regenerative response to liver resection [17]. A limited tuning to human time scales was performed using extremely low amount of clinical data available on liver volume at more than a year after the surgical resection [7, 8]. Hence, it is questionable whether the findings from the simulation and analysis of the rodent-based models are applicable to the human condition. Recently, time series data on volumetric changes in liver have become available in a relatively large number of patients that have undergone different levels of surgical resection of the liver [18]. This data on the dynamic changes in liver volume, presumably correlated with the tissue mass, enables an opportunity to tune the network model to match the time scales relevant to the human liver regeneration, and analyze the resultant computational model for different “virtual patients” based on their clinical tests and the effect on the regenerative outcome.

In this study, we build on our previous computational model of rat liver regeneration [8] and tune the parameters to the human time scale, by utilizing select data from a recently available clinical data set [18]. We initially tuned the model parameters based on constrained optimization. Subsequently, we considered a range of parameter variations corresponding to different “virtual patients” to analyze the model-predicted response dynamics, and evaluated the directional influence of each of the parameters on the liver mass outcome following resection. Through this approach, we identified a subset of virtual clinical cohort of patients that exhibit recovery or failure, depending on the corresponding parameter values. We analyzed sensitive parameters individually and in combination to delineate the parameter subspaces that correspond to three response modes - full recovery, partial recovery, failure. We utilized phase plane analysis [19, 20] to compute a threshold of resection that separates the recovery versus failure response modes exploiting their basins of attraction. We assessed how this failure threshold varies for different “virtual patients”. Phase plane analysis demonstrated that the variation in response of this cohort of patients yields distinct phase planes with either one sink of failure or two sinks corresponding to recovery and failure. This patient-specific parameter-dependent shift in the multiplicity of the system may help with decision making on whether liver surgery is a viable option in certain cases. For the case of two sinks, recovery and failure, our model-based analysis suggests the level of resection that is likely to be safe.

## Results

### Tuning the model for human time scale of response based on patient data

**Fig. 1:**
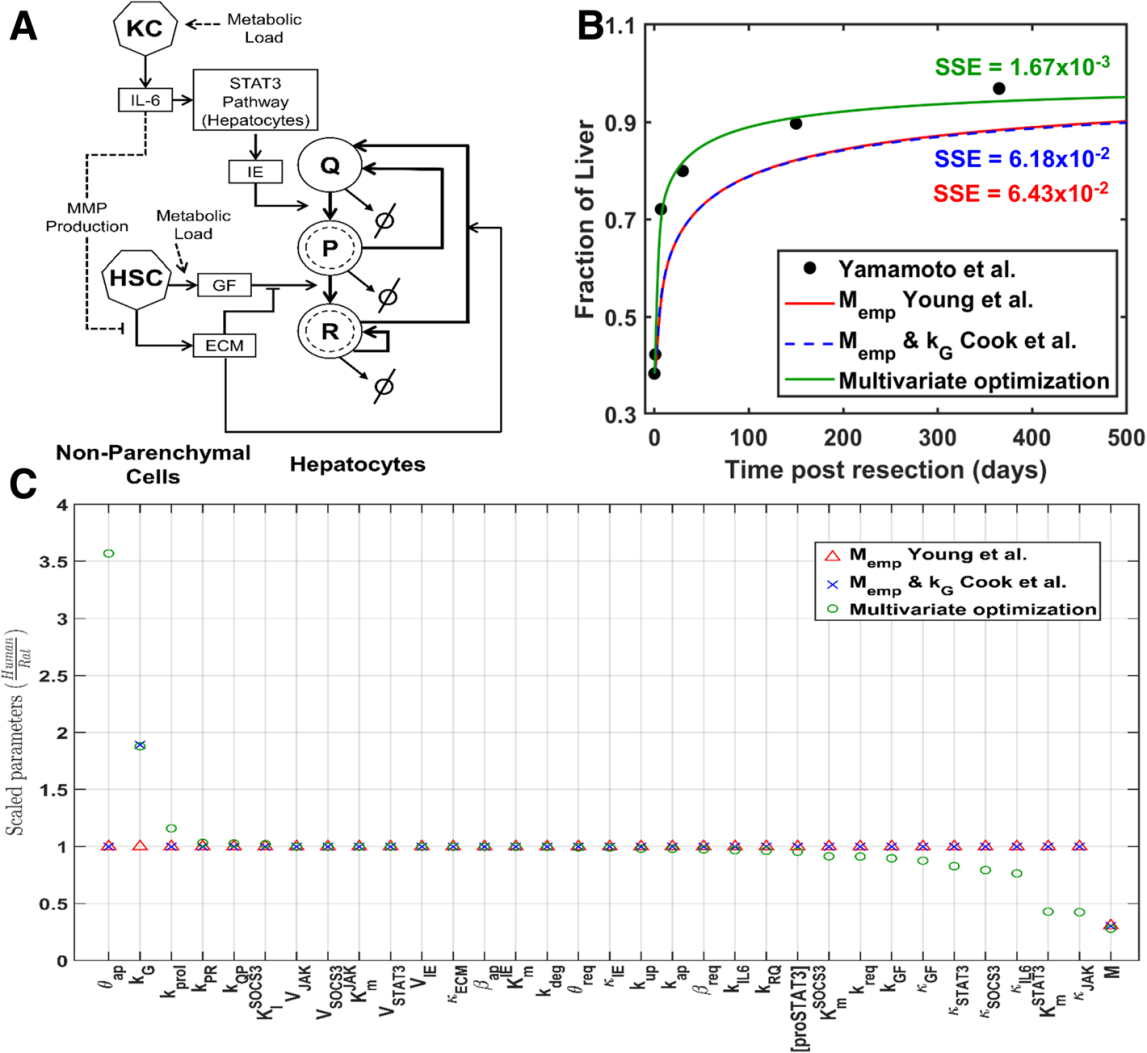
**a** Network scheme of the liver regeneration model. **b** Comparison of approaches for translating the model across species. Regeneration profile corresponding to patient ID71 [18] obtained via the three approaches: empirically scaling only the metabolic load based on species body mass [21], scaling metabolic load based on body mass along with modifying the relative cell mass growth constant (k_G_) to 6.5675e-4 for human case [8], and present multivariate optimization approach where all the model parameters were allowed to be tuned. **c** Relative variation of all the 33 parameters of the model scaled by Cook et al. [8] parameters for rat

The computational model employed here is based on the network model of Cook et al. [8] (Fig. 1a). In this scheme, liver regeneration response to partial hepatectomy (PHx) is driven by the metabolic load, which is considered as an organ-scale parameter. Increase in metabolic load per unit of liver mass following PHx (due to reduction in remnant liver mass) elicits a cascade of signals leading to the activation of hepatic non-parenchymal cells (Kupffer cells and hepatic stellate cells (HSCs)). Activated Kupffer cells release IL-6 which activates JAK-STAT pathway in hepatocytes. This results in the production of immediate early genes (IE) which regulates the priming of hepatocytes. Concurrent remodeling of extracellular matrix (ECM) by MMPs secreted by HSCs releases matrix bound growth factors, such as HGF (hepatocyte growth factor), produced by HSCs and liver sinusoidal endothelial cells and dispersed in the ECM in an inactive form. Binding of GFs to surface receptors of primed hepatocytes induces intracellular MAPK cascade activation and progression of hepatocytes through the cell cycle.

Although the Cook et al. [8] model characterizes the important attributes of rodent liver regeneration, it fails to capture the liver regeneration time scale accurately in humans, largely attributed to the lack of availability of human time series data when the original model was tuned. We used our modified model and estimated the human time scale relevant parameters using patient data from Yamamoto et al. [18] (see Methods). We utilized liver volume time series data from a set of 27 patients that showed recovery following resection, with sufficient number of time points in the regeneration profile to permit model tuning and analysis of dynamics. We pursued an optimization-based approach that allowed variations in all the parameters to identify the tuning with best fit to the clinical data. We compared the results of our parameter tuning approach to that of Young et al. [21] and Cook et al. [8] approaches for translating the model parameters across species based on body mass. In case of Young et al. [21], only the metabolic load (M) is tuned, whereas Cook et al. [8] approach also modified the relative cell mass growth constant (k_G_). We applied the three parameter tuning approaches to each of the 27 patient time series data sets and compared the results.

An illustrative case of model-derived liver regeneration profiles comparing the three parameter tuning approaches is shown in Fig. 1b. In this case (patient ID 71), the two regeneration profiles derived from scaling the metabolic load qualitatively capture the clinically observed trend in liver recovery, but do not match the observed time scale and long-term recovery level. By contrast, the multi-parameter optimization approach achieved a better fit to the observed dynamics and long-term recovery level with a lower residual error. The optimization approach resulted in variations in multiple parameters, five of which were significantly scaled in translating the rodent model to the human case (Fig. 1c). Even when optimizing all the parameters for best fit to data, M and k_G_ were two of the top three parameters that were altered from baseline rat-based parameters. Interestingly, the metabolic load value was similar across the three approaches for translating the rat model to the human case (Fig. 1c). The cell growth parameter was also tuned similarly between Cook et al. [8] approach and the multiparameter optimization (Fig. 1c). In the latter case, three additional parameters were varied in order to achieve the best fit: cell death sensitivity parameter θ_ap_was higher, and immediate early signaling parameters, ${\mathrm{K}}_m^{STAT3}$ and κ_JAK_ were lower in the human case compared to the rodent model.

We compared the optimization-derived and the metabolic scaling-based parameter values across the 27 patients. In the case of multiparameter optimization, the parameters were tuned individually to each of the 27 time series profiles. In the case of metabolic scaling-based tuning, we utilized the patient-specific BMI and derived body mass based on the average height of Japanese male and female, as appropriate (see Methods). For the Cook et al. [8] case, the relative cell mass growth constant (k_G_) was modified to 6.5675e-4, as per Cook et al. [8]. Our results indicate that the metabolic scaling parameter was tuned very similarly across all three approaches, yet with dissimilar fit to data as noted by the differences in the residual error (Additional file 1: Table S1). We observe that the metabolic scaling approaches of Young et al. [21] and Cook et al. [8] capture the regeneration dynamics only in a subset of the cases, and the multiparameter optimization improved the fit to the patient time series data in most cases (Additional file 1: Table S1). This improved fit in a subset of the patient cases was achieved by variations in additional parameters. A representative case of the model fit to data where the optimization-based approach yielded similar parameters as the metabolic scaling approach is shown in Additional file 2: Figure S1. This is in contrast to the ID 71 case shown in Fig. 1, where several parameters were altered to translate the model from rat to human case.

**Fig. 2:**
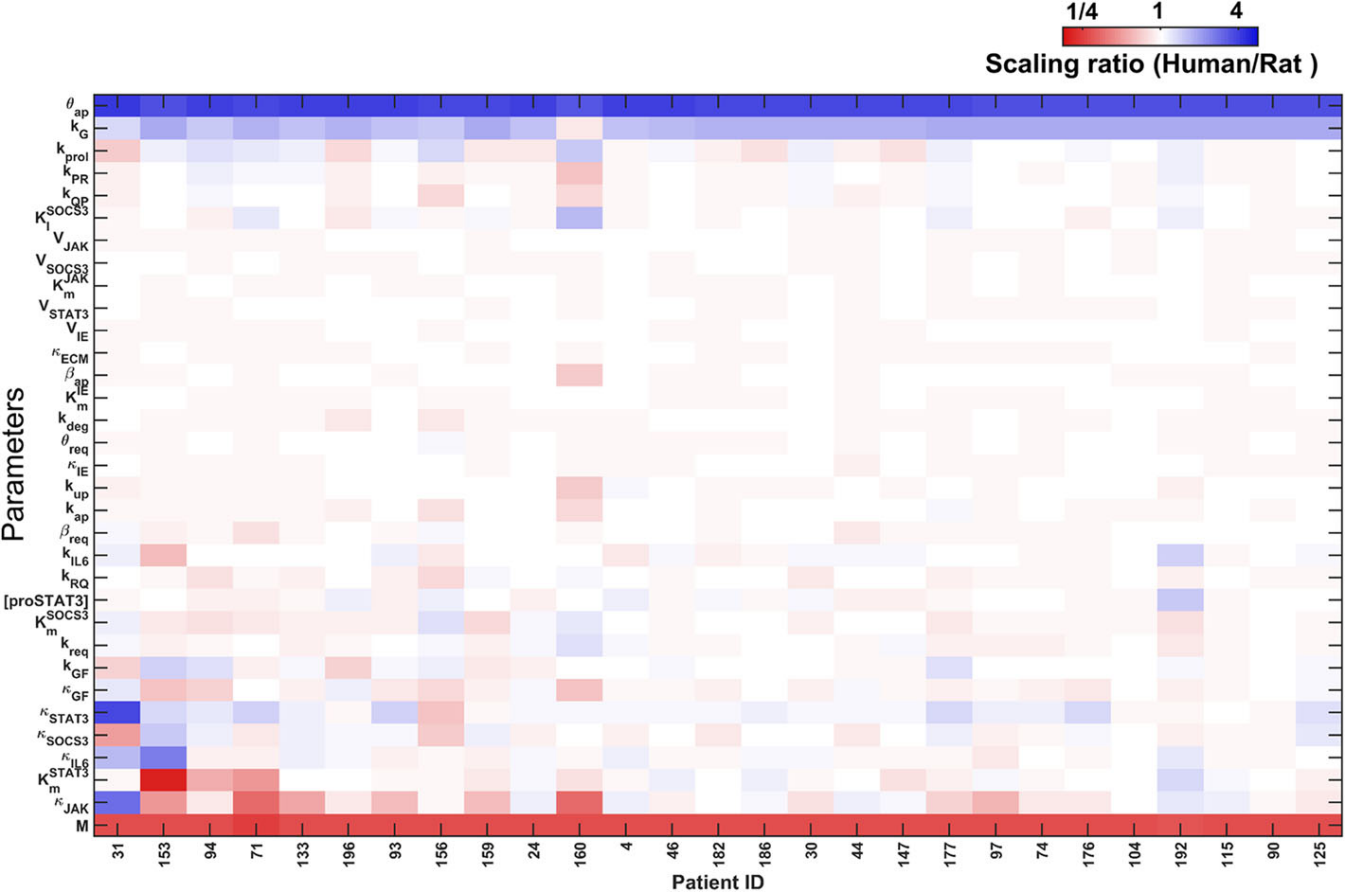
Heatmap of optimized model parameters scaled to that of rat parameters from Cook et al. [8]. The parameters metabolic load, cell death sensitivity and relative cell mass growth constant show significant difference across species, with relatively consistent scaling needed across all patients. Other parameters were more variable across patients and likely represent patient-specific liver cell-intrinsic differences

Comparison of the optimization-based parameters across patients show that metabolic load (M) and the relative cell mass growth parameter (k_G_), and the cell death sensitivity parameter (θ_ap_) were tuned with consistent up- or down-scaling in all the patients (Fig. 2). Additional parameters were not altered consistently across all the patients, suggesting patient-specific tuning may be required in some cases, to go beyond the overall metabolic scaling and adjustment of cell death sensitivity, to better account for the human liver regeneration dynamics. We evaluated whether the parameters correlated with the available clinical data, and found very few significant pair-wise correlations between the parameter values and clinical variables (Additional file 3: Figure S2). Hence, it is not immediately clear if the patient-specific hepatocyte-intrinsic parameters can be derived from the gross clinical measurements available presently. We note caution in generalizing these results to larger populations, considering the low number of patients analyzed in the present data set. We analyzed the correlations between the optimization-based parameters to evaluate whether certain parameters were constrained together when tuning the model across patients. The results shown in Additional file 4: Figure S3 illustrate that majority of the parameters were not correlated with each other (Bonferroni-adjusted *p* < 1e-5) at low correlation values (absolute correlation < 0.5). This result suggests that the variability across patients may not be highly constrained by correlated variations in the underlying parameters. While additional analysis can be performed by fractionating the data into groups, e.g., sex-specific, or age-specific, etc., we caution that the present sample set may be too low to generalize the results to a larger population.

In summary, our results point to the need for tuning the cell death sensitivity parameter (θ_ap_) in addition to tuning the metabolic scaling and the relative cell mass growth parameters. These differences in model tuning may be attributable to the differences in the temporal resolution of the time series data that were used to derive Young et al. [21] and Cook et al. [8] results versus the Yamamoto et al. [18] data we employed in the present study. We conducted subsequent analysis using the parameter values derived from optimizing all the parameters shown in Table 1.

**Table 1:** Optimal values of the parameters corresponding to patient ID71 from Yamamoto et al. [18]

Parameter	Optimized value	Parameter	Optimized value
M	5.8206	k_deg_	6.9843
k_IL6_	1.4528	κ_ECM_	32.9924
κ_IL6_	0.6878	k_GF_	0.1014
V_JAK_	20,000	κ_GF_	0.2016
${\mathrm{K}}_{\mathrm{M}}^{\mathrm{JAK}}$	9999.9999	k_up_	0.0589
κ_JAK_	0.1695	k_QP_	0.0072
[proSTAT3]	1.9108	k_PR_	0.0045
V_ST3_	749.9994	k_RQ_	0.0520
${\mathrm{K}}_{\mathrm{M}}^{\mathrm{ST}3}$	0.1715	k_prol_	0.0232
κ_ST3_	0.0828	k_req_	0.0912
V_SOCS3_	24,000.0000	θ_req_	7.9493
${\mathrm{K}}_{\mathrm{M}}^{\mathrm{SOCS}3}$	0.0006	β_req_	2.9285
κ_SOCS3_	0.3173	k_ap_	0.0982
${\mathrm{K}}_{\mathrm{I}}^{\mathrm{SOCS}3}$	0.0153	θ_ap_	0.0321
V_IE_	249.9992	β_ap_	0.0045
${\mathrm{K}}_{\mathrm{M}}^{\mathrm{IE}}$	17.9736	k_G_	0.0007
κ_IE_	4.9595		

### Analysis of virtual patients by varying individual parameters

We analyzed the individual effect of all the 33 model parameters on the long-term liver mass recovery fraction after two and half years post hepatectomy. Starting with the human time scale optimized parameter vector from the previous section, we varied one parameter at a time to develop a distribution of “virtual patients”. We simulated the liver regeneration profiles for each patient over the range of each varied parameter, while keeping the remaining parameters at their optimized values. We use the term “virtual patient” since all the parameter values are not patient-specific, but rather that these parameters take values within biologically reasonable bounds around the human time scale optimized value. We use this approach rather than the local sensitivity analysis in which a parameter is only marginally varied one at a time, since we are interested in the directionality of the influence of each parameter on the liver mass outcome over a wide range of parameter values.

The model-predicted temporal profiles of liver mass recovery fraction were used to classify the parameters into two broad categories – those with a consistent effect on liver mass recovery fraction over the full range of parameter variation, and those that could lead to either mass recovery or liver failure, depending on the parameter value. The parameters that always lead to liver recovery with varying level of mass outcome (i.e., “gain” controllers) are further divided into two groups, denoted as sensitive and insensitive parameters. Sensitive parameters are of two types, one that helps in improving the recovery of liver as the parameter value increases (such as k_IL6_), and the other type that slows down the liver recovery upon increasing the parameter value (e.g., κ_IL6_). The insensitive parameters do not impact the liver mass recovery significantly irrespective of the parameter value within the varied range (e.g., k_ap_). The parameters that can lead to both recovery and failure, depending on the parameter value, were also further divided into two groups, denoted as sensitive and insensitive parameters. The sensitive parameters were M (metabolic load) and β_ap_ (cell death sensitivity), and the insensitive parameter was θ_ap_ (cell death threshold). Table 2 shows the different class of parameters with the model parameters in their respective categories.

**Table 2:** Model parameters categorized according to the effect on liver response profile, leading to recovery alone, or causing either recovery or failure depending on the parameter value

Only recovery			Both recovery and failure	
Sensitive		Insensitive	Sensitive	Insensitive
Improves recovery	Decelerate recovery			
$$ {\displaystyle \begin{array}{l}{k}_{IL6},\left[ prosSTAT3\right],\\ {}{\kappa}_{SOCS3},{K}_I^{SOCS3},{V}_{IE},\\ {}{k}_{\mathrm{deg}},{k}_{GF},{k}_{QP},{k}_{PR},\\ {}{k}_{prol},{\beta}_{req},{k}_G\end{array}} $$	$$ {\displaystyle \begin{array}{l}{\kappa}_{IL6},{K}_M^{JAK},{\kappa}_{JAK},\\ {}{K}_M^{ST3},{V}_{SOCS3},\\ {}{K}_M^{SOCS3},{K}_M^{IE},{\kappa}_{IE},\\ {}{\kappa}_{ECM},{\kappa}_{GF},{k}_{up},\\ {}{k}_{RQ},{k}_{req}\end{array}} $$	$$ {\displaystyle \begin{array}{l}{V}_{JAK},{V}_{ST3},{\kappa}_{ST3},\\ {}\;{\theta}_{req},{k}_{ap}\end{array}} $$	*M*, *β*_*ap*_	*θ*_*ap*_

**Fig. 3:**
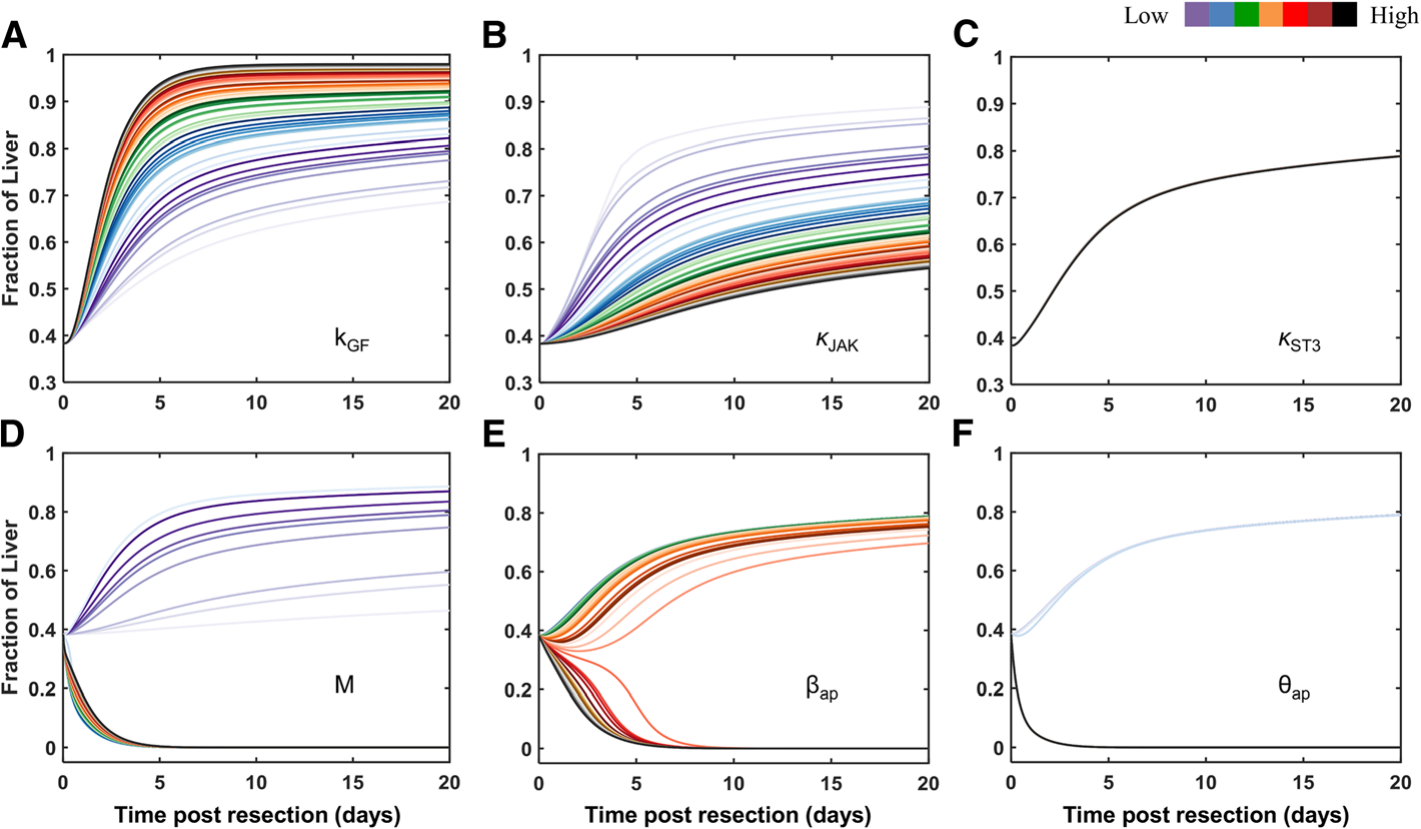
Regeneration profile showing directional change in the recovery for representative parameters belonging to different classes. Parameter value increases in the order of color scheme shown in the color bar. **a**, **b**, **c** Only recovery occurs, which improves with increasing k_GF_ (**a**), deteriorates with increasing κ_JAK_ (**b**), and is insensitive to increase in κ_ST3_ (**c**). **d**, **e**, **f** Both recovery and failure occur, and are sensitive to changes in M (**d**) and β_ap_ (**e**), insensitive to changes in θ_ap_

The regeneration profiles for each class of parameters are shown in Fig. 3. We observed that while variations in either k_GF_ (Fig. 3a) or κ_JAK_ (Fig. 3b) result in liver recovery, these parameters exert opposing directional influence on the liver recovery. κ_ST3_ (Fig. 3c) is the insensitive parameter that leads to only recovery with no directional influence on the model output. Metabolic load M and the cell death parameter β_ap_ were the sensitive parameters that can result in either liver failure or recovery depending on the parameter values. Increasing the value of M, starting at a low value, supported liver recovery outcome up to a threshold (Fig. 3d). Increasing M beyond a threshold value resulted in liver failure in our simulations. Similarly, our simulations suggest that low β_ap_ results in liver recovery, which decelerates recovery with increasing β_ap_ and leads to liver failure at higher values of β_ap_ (Fig. 3e). θ_ap_ leads to liver recovery at low values and results in failure beyond a certain threshold. However, there is no directional influence of θ_ap_ on the liver mass fraction across the threshold.

We sought to characterize [8] the differences between the present study and Cook et al. model [8] in terms of the differences in parametric sensitivity. We performed a similar analysis of directional influence of all the model parameters on liver regeneration outcome in a cohort of virtual patients using Cook et al. model [8]. Our results indicate that majority of the parameters showed similar effects on the liver response in Cook et al. model [8] and in the present model (Additional file 5: Table S2). Due to numerical difficulties in integrating the Cook et al. model [8] for certain parameter values, the effects of variation in the parameters M, k_IL6_, k_prol_, θ_ap_, β_ap_ were evaluated in a more restricted range than the present model. These issues were overcome in the present model by consideration of senescent fraction in accounting for overall tissue mass (parameter ε in model equations below). [8]

Owing to the high sensitivity of our model to M and β_ap_ (Fig. 2d-e) as well as physiological relevance of increased hepatocellular metabolic activity and cell death (linked to M and β_ap_, respectively) in the context of liver regeneration [22], we examined the effect of these parameters on the liver mass fraction response to resection in a cohort of virtual patients.

### Shift in the regeneration modes of virtual patients with varying metabolic load and cell death sensitivity for different levels of resection

We analyzed the impact of intrinsic perioperative factors, metabolic load and cell death sensitivity, as well as the extrinsic factor of different levels of resection, on the regeneration modes of a cohort of virtual patients. We examined the model-predicted temporal response of an individual patient’s liver mass post hepatectomy by focusing on the effect of variation in specific parameters that can cause liver failure or recovery, depending on the parameter values. Our parameter correlation analysis indicated that the metabolic load (M), cell death sensitivity (β_ap_) parameters did not vary in a correlated manner with any of the model parameters across the patient data set (Additional file 4: Figure S3). Hence, in the present manuscript, we generate and analyze virtual patient cohorts that span the entire range for the subset of parameters considered without excluding any parameter subspaces, following similar practice in literature [23].

**Fig. 4:**
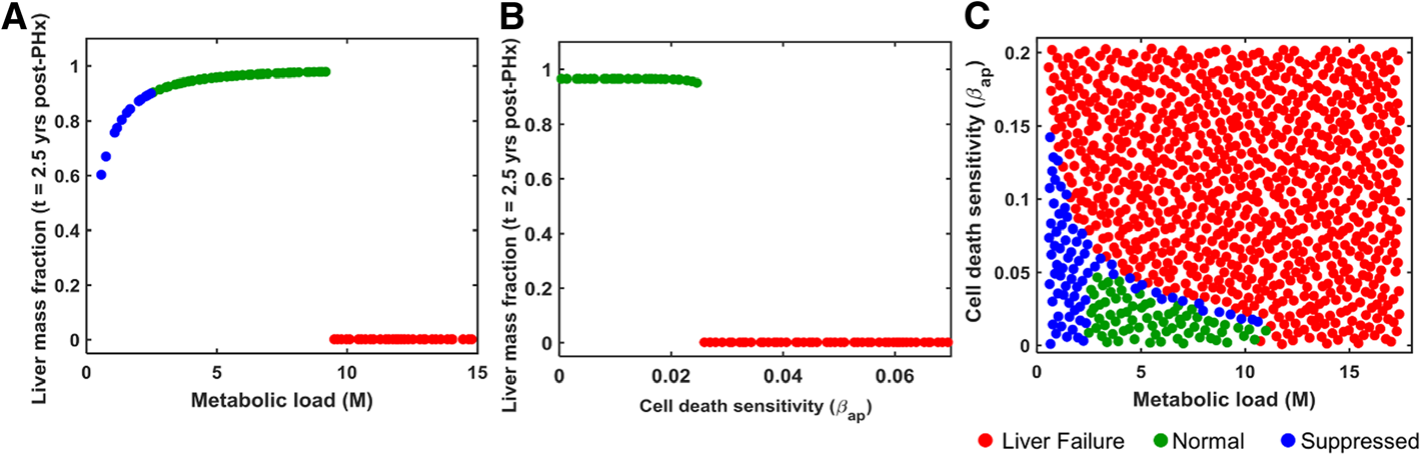
**a** Effect of metabolic load on long-term liver mass fraction. **b** Effect of cell death sensitivity parameter on long-term liver mass fraction. **c** Effect of simultaneous variation in both the metabolic load and cell death sensitivity parameters on liver recovery after 2/3^rd^ PHx. Each marker represents one virtual patient considered in the simulation

We studied the regeneration modes of normal, suppressed and liver failure in virtual patients. Initially, we varied the two parameters (M and β_ap_) individually using a Sobol sample of size 1000, with the remaining 32 parameters fixed at the human time scale optimized values (Table 1). Simulations indicate that patients with low metabolic load show suppressed growth (Fig. 4a), as the stimulus for regeneration is not strong enough to trigger proliferation in all the cells. By contrast, high levels of metabolic load leads to liver failure due to excessive injury and resulting cell death. These results predict that in order to achieve normal liver regeneration and growth, the metabolic load should neither be too high leading to liver failure, nor be too low resulting in suppressed growth. Similar analysis performed for the cell death sensitivity parameter β_ap_ predicted that lower value of β_ap_ results in normal growth, whereas higher values lead to liver failure due to excessive cell death (Fig. 4b).

Subsequently, we simulated a cohort of virtual patients to analyze the combined effect of variation in M as well as β_ap_ on liver regeneration. We generated a cohort of 1000 virtual patients based on simultaneous variations in the parameters, M and β_ap_, while the remaining 31 parameters were fixed at their optimized values. Simulations revealed that the parameter space can be partitioned into three distinct regions corresponding to different response modes (Fig. 4c). At low values of both the critical parameters, resection leads to a suppressed response mode. At high values of either or both of these critical parameters, resection leads to liver failure. It is likely that liver failure occurs in the case of high metabolic load as the remnant cells are unable to meet the high functional requirements. The intermediate region corresponds to normal growth enveloped by the suppressed growth mode. These model predictions indicate that complete liver mass recovery requires a balance between the two intrinsic perioperative factors (metabolic load and cell death sensitivity). To evaluate the impact of these intrinsic perioperative factors on the liver regeneration in rats we analyzed the combined effect of variations in metabolic load and cell death sensitivity (M and β_ap_) in our model for 33.3% hepatectomy with the rat specific parameters from Cook et al. [8]. We found that the distribution of the suppressed, normal and failure response modes in the parameter space of M and β_ap_ is similar in the rat to that in human case, even though the specific values of these parameters are different for the two species (Additional file 6: Figure S4). Suppressed mode was observed for low metabolic load and cell death sensitivity, while failure mode occurred at the other extreme of high values for these factors. The results suggest that normal recovery mode likely depends on a balance between metabolic load and cell death sensitivity in both rat and human liver resection scenarios.

**Fig. 5:**
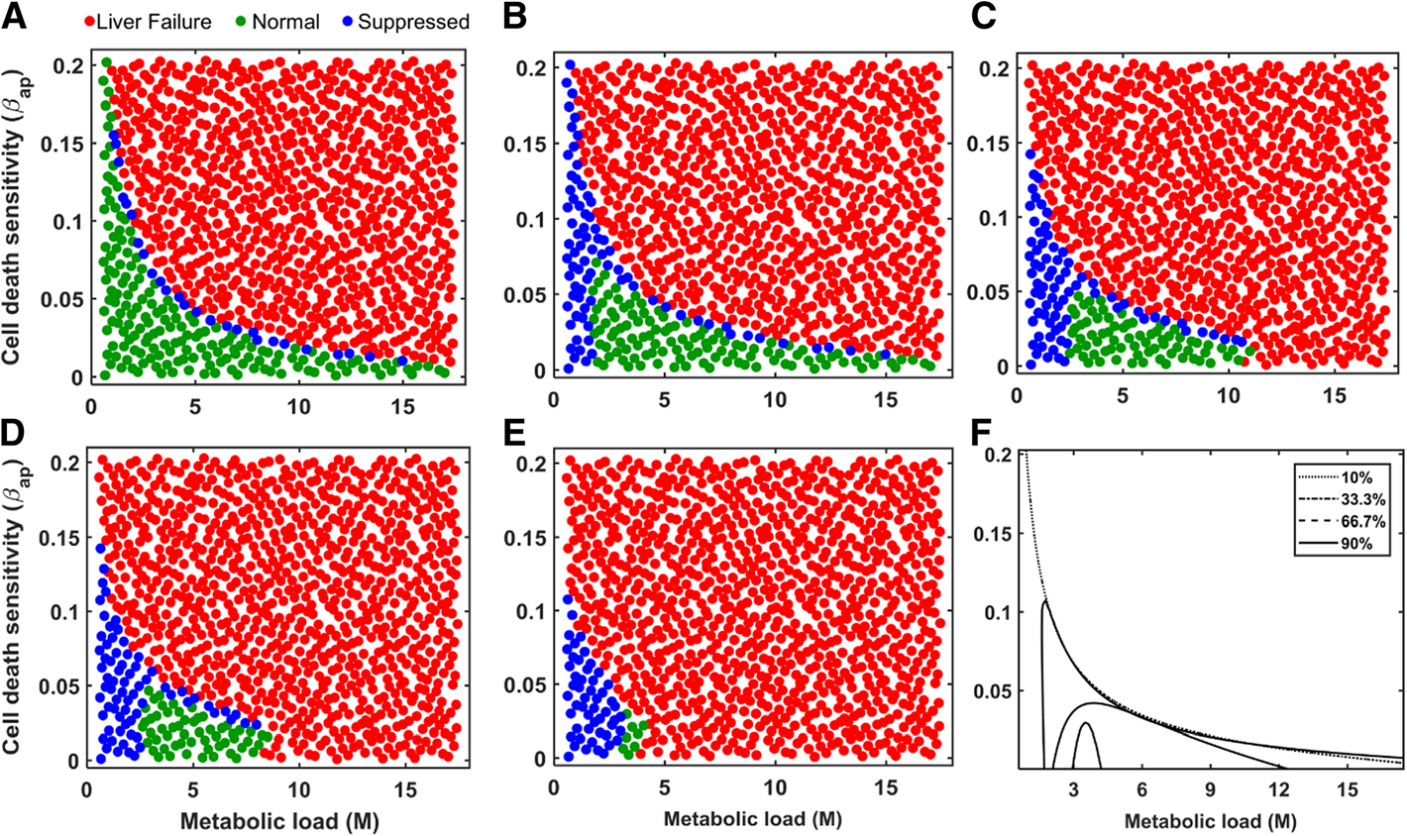
Parameter space depicting regions of distinct regeneration modes for different levels of resection: **a** 10% PH; **b** 33.33% PH; **c** 66.67% PH; **d** 75% PH; **e** 90% PH. Each marker represents a virtual patient. **f** Changes in the extent of parameter space corresponding to the full mass recovery with varying levels of resection

We next analyzed the impact of an extrinsic perioperative condition - different levels of resection - on the regeneration modes of the same cohort of virtual patients. We considered a cohort of 1000 virtual patients based on simultaneous variations in both metabolic load and cell death sensitivity parameters, for different levels of resection: 10, 33.3, 66.7, 75 and 90% (Fig. 5a-e), while holding the remaining 31 model parameters fixed at the optimized parameter values. Model simulations indicate that for low level of resection, the parameter subspace corresponding to the suppressed mode sharply separates the normal growth and liver failure regions. With an increase in the resection level, the suppressed mode progressively spreads towards the virtual patients with lower metabolic load and envelops the normal growth region. This qualitative change of the suppressed region is such that a one-third resection results in suppressed growth in a virtual patient with low metabolic load, irrespective of the cell death sensitivity. However, at increased resection levels, the extent of parameter subspace corresponding to the suppressed regeneration mode decreases with a corresponding increase in liver failure cases. In addition, the extent of parameter subspace corresponding to full recovery shrinks (Fig. 5f), eventually into a very small region for the 90% resection case which may not be easy to attain in a real scenario. This dynamic change of different response regions in the parameter space results in two distinct types of transitions towards liver failure: (1) First type of transition corresponds to a shift from normal/suppressed mode to liver failure, which may be similar to the response of patients with chronic liver disease, resulting in impaired regeneration. (2) The second type corresponds to the direct transition from normal recovery to liver failure mode, which may have parallels to the response in an acute liver injury scenario. In the next section, we analyzed the effect of these two critical parameters on the threshold of liver failure.

### Determining the safe level of resection based on the metabolic load (M), an intrinsic perioperative factor

**Fig. 6:**
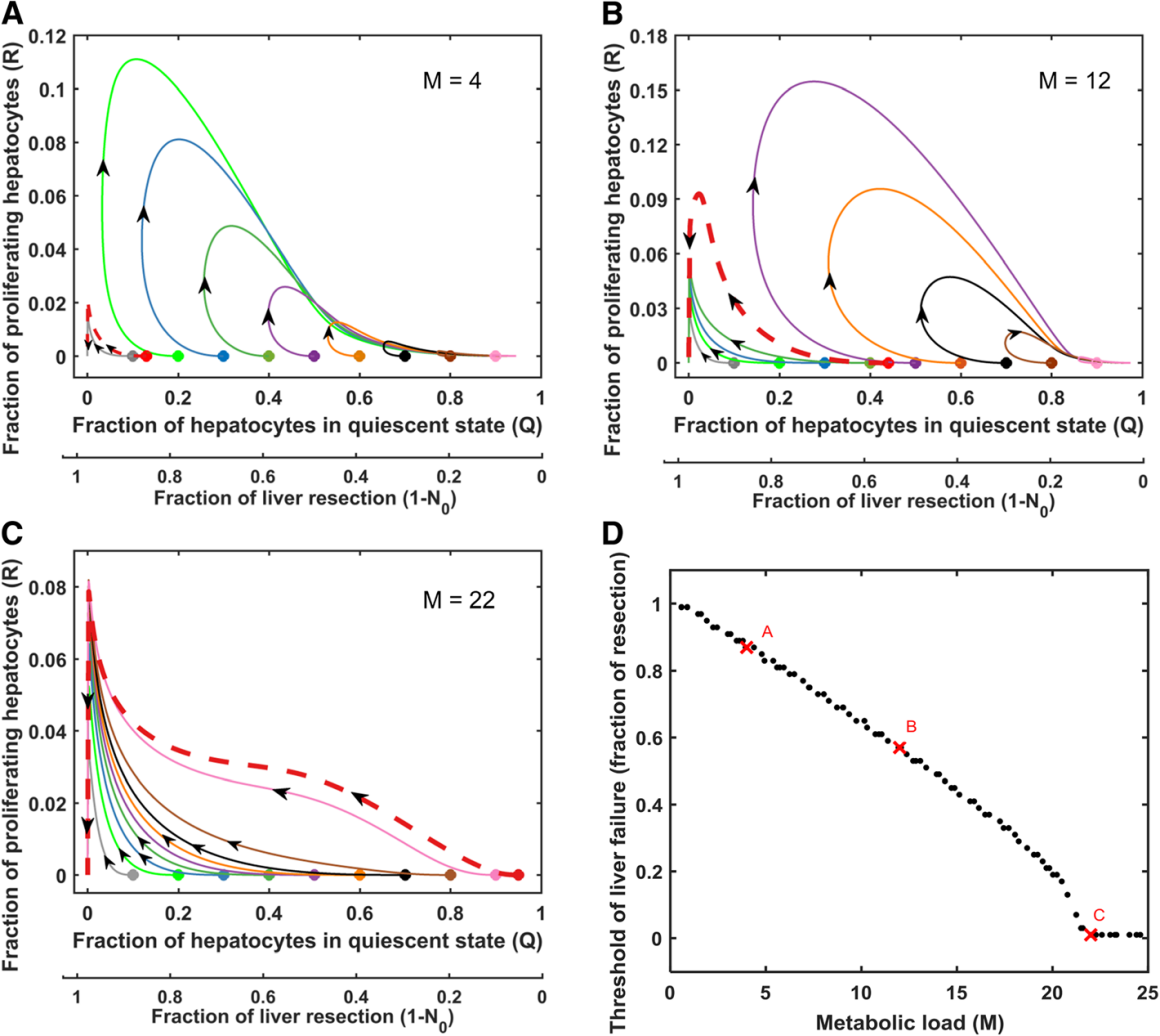
Phase portrait for quiescent (Q) and replicating (R) cell fractions with varying levels of metabolic load parameter M. All other parameters were set to the optimal levels given in Table 1 The filled circle markers in **a**-**c** represent different levels of resection. The red dashed curves represent trajectories for the critical level of resection at and above which failure occurs. **a** M = 4, yields a threshold of liver failure at 87% resection. **b** M = 12, yields a threshold of liver failure at 56% resection. **c** M = 22, for which there is no safe level of resection. **d** Influence of metabolic load on the threshold of liver failure. Red cross markers in panel **d** represent the threshold of failure for the corresponding phase planes **a**-**c**

We pursued the dynamical systems approach of phase portrait analysis to evaluate the safe level of resection in a cohort of virtual patients that differ in the level of metabolic load, an intrinsic perioperative factor. We examined the effect of metabolic load on the temporal trajectory of the balance of quiescent versus regenerating hepatocytes for different levels of resection (Fig. 6). Each phase plane corresponds to an individual virtual patient with a specific metabolic load parameter, M. Each trajectory in the phase plane represents the evolution of liver mass with time for a given level of resection.

The phase portrait for the virtual patients under study captures the two attractors of liver failure and liver recovery (Fig. 6a). The phase plane analysis demonstrated that the trajectories for high levels of resection progress towards the liver failure attractor, whereas trajectories for low levels of resection converge to the attractor of liver recovery. The phase plane shows a clear demarcation between the safe and the unsafe level of resection for liver surgery. Figure 6a-c shows the phase plane for increasing values of metabolic load. The results indicate that the span of the attractor for liver recovery progressively decreases with increasing metabolic load, and finally vanishes, leading to liver failure irrespective of the level of resection. This suggests a change in the multiplicity of the system, which may have clinical implications in considering the surgical options. We utilized the phase plane analysis to examine the threshold of liver failure for a given virtual patient. Our analysis indicates an inverse monotonic relationship between threshold of liver failure and metabolic load, such that higher the metabolic load lower is the level of maximum resection that can still lead to recovery, i.e., higher requirement for safe level of remnant liver mass post resection (Fig. 6d).

In parallel, we analyzed the effect of the intrinsic perioperative factor of cell death sensitivity (β_ap_) on the threshold of failure. Our results indicate that cell death sensitivity has a significant effect on the safe level of resection (Additional file 7: Figure S5A-C), with a similar inverse monotonic relationship between the level of cell death sensitivity and threshold of failure (Additional file 7: Figure S5D).

### Determining the safe level of resection based on variations in both metabolic load and cell death sensitivity (M and β_ap_)

Thus far, we analyzed the threshold of liver failure (conversely, the safe level of resection) in a cohort of virtual patients based on individual variations in either metabolic load (M) or cell death sensitivity (β_ap_). Both these parameters had a similar effect on the threshold of failure of the virtual patients. In this section, we investigated how the threshold of failure changes in virtual patients based on simultaneous variations in these two intrinsic perioperative parameters. We simulated the dynamic model by varying the two parameters (M and β_ap_) using a Sobol sample of size 50 × 50, and the remaining parameters were fixed at human optimized value for two-thirds resection (Fig. 1b; Table 1). Sobol sampling of size 50 × 50 corresponds to metabolic load and cell death sensitivity such that each value of metabolic load is paired with all the values of cell death sensitivity and vice-versa. This approach yielded a cohort of 2500 virtual patients.

**Fig. 7:**
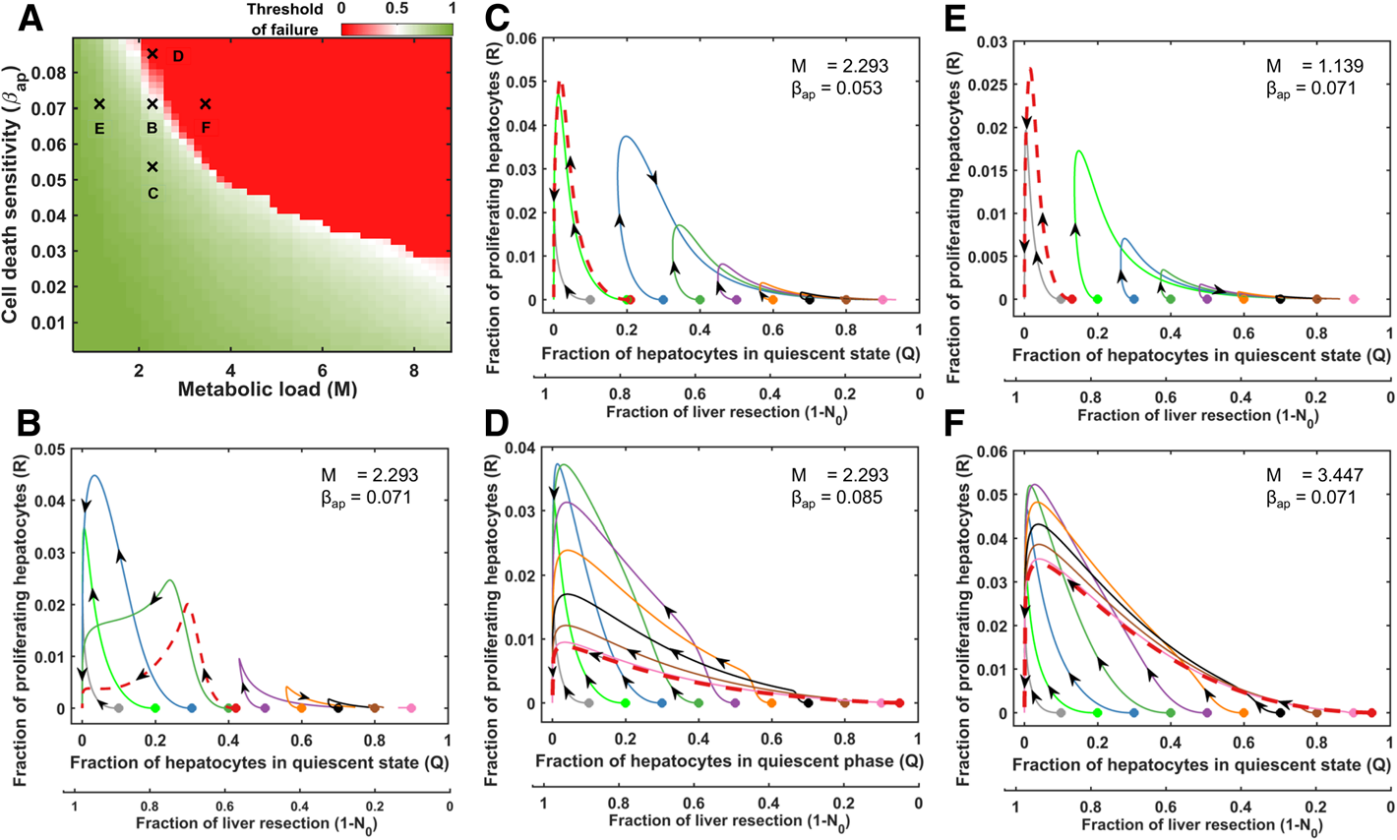
**a** Heatmap showing influence of metabolic load and cell death sensitivity parameters on the threshold of liver failure in terms of fraction of resection that is safe. Black cross markers represent the virtual patients for the corresponding phase portraits in panels **b**-**f**. **b** Phase plane for metabolic load (M) = 2.293 and cell death sensitivity (β_ap_) = 0.071, yielding a threshold of failure at 58% resection. This scenario corresponds to the critical transition from high threshold to low threshold of liver failure on the heatmap in panel **a**. **c** M = 2.293 and β_ap_ = 0.053, yields a threshold of failure at 79% resection. **d** M = 2.293 and β_ap_ = 0.085, yields a virtual patient for whom all levels of resection lead to failure. **e** M = 1.139 and β_ap_ = 0.071, yields a threshold of failure of 87%. **f** M = 3.447 and β_ap_ = 0.071, yields a virtual patient for whom all levels of resection resulted in a failure. The circular markers in the phase portraits represent different levels of resection and the red curves denote the critical level of resection above which the system progresses towards the failure mode

Based on the simulation results from 2500 virtual patients, we developed a map of the threshold of failure as a function of metabolic M and cell death sensitivity β_ap_ (Fig. 7a). Low values of both the parameters resulted in a high threshold of failure (i.e., larger resections are safe), and conversely high values of these two parameters diminished the threshold of failure to low levels (i.e., only small resections remain safe). The Fig. 7a map shows a clear separatrix between the two regions corresponding to recovery and failure. We analyzed the phase planes corresponding to distinct locations in the Fig. 7a map. The phase plane corresponding to a location on the separatrix is shown in Fig. 7b. For this case, a resection of up to 58% led to liver recovery. By contrast, the phase portrait for a lower level of cell death sensitivity parameter, at same metabolic load as in Fig. 7b, yielded a threshold of failure of 79% (Fig. 7c). Increasing the cell death sensitivity for the same level of metabolic load as in Fig. 7b, shifted the system into the zone without a safe level of resection (Fig. 7d). Correspondingly, holding the cell death sensitivity at the same level as in Fig. 7b, and altering the metabolic load shifted the threshold of failure between the two regions demarcated by the separatrix. The phase plane of the virtual patient with the lower metabolic load yielded a threshold of failure at 87% resection (Fig. 7e), whereas the virtual patient with the higher metabolic load exhibited liver failure for even a small level of resection (Fig. 7f). These results demonstrate that, the phase portrait with a high threshold of failure (green zone of the Fig. 7a map) contains two attractors, corresponding to liver failure and liver recovery modes. By contrast, the phase plane with a low threshold of failure (red zone of Fig. 7a map) contained only one attractor, corresponding to that of liver failure. These results demonstrate the utility of the phase transition map for determining the safe level of resection for a given patient.

## Discussion

We started with a quantitative model of liver regeneration response to resection and fine-tuned the parameters to account for a normal liver recovery profile at human-relevant time scales. We built on this initial simulation and analyzed the distribution and modes of response of a virtual patient cohort to varying level of resection, which potentially account for differences due to disease etiology, patient demographics, and perioperative conditions. Notably, the range of parameter variation covered a full span of an individual virtual patient’s potential response along three distinct modes: accelerated growth, slower recovery, and failure. Our approach differs from that of a population of models (POM) approach [24], in which the objective is to account for the distribution of responses in clinical data, and the response of a virtual patient is accepted or rejected based on a specified tolerance limit. By contrast, our approach is targeted at characterizing the entire range of responses to analyze the distinct modes of response across all virtual patients. Such an unbiased approach has been pursued in other studies with informative results on parameter subspaces that distinguish qualitatively different patient responses [23]. Our Sobol sampling-based wide range of simulations led us to identify subsets of critical parameters and their combinations that govern the transitions in the response of virtual patients to varying levels of resection. Our approach to accounting for dynamics of human liver regeneration response to resection is different from that of Yamamoto et al. [18] study from which we utilized the liver volumetric data to tune the computational model parameters. Yamamoto et al. [18] model was based on modification of a logistic model whose response is largely governed by the sign of the initial rate of response to resection (positive value leading to recovery, and negative value leading to failure). This simplified representation allowed the development of a discriminant function to correlate the rate of liver regeneration to the pre- and perioperative clinical factors, and then predicted the outcome based on a binary classification of the initial rate of liver regeneration being positive or negative. By contrast, we utilized a multi-scale network model that contains a relatively more detailed representation of molecular interactions and cellular functional states, and tuned the parameters of the model to account for the observed timescales of human liver regeneration. We analyzed the influence of key parameters on the outcome based on the level of resection, and examined the quantitative relationship between variations in two key parameters and the threshold of failure.

Our study considered a cohort of virtual patients based on two critical parameters: metabolic load and cell death sensitivity. These two model-predicted critical parameters are perioperative factors and have the potential to be estimated from patient-specific clinical information. For instance, metabolic load can be empirically related to mass, body mass index [25], and further modified based on other factors such as age, gender, etc. of the patient [26, 27]. We expect that cell death sensitivity can be related to disease etiology, patient’s medical history, and perioperative conditions such as blood loss (Yamamoto et al. [18]). It is reasonable to expect that certain patients may be more sensitive to injury than others depending on the advanced versus earlier stage of the underlying liver disease. In addition, aging also has a significant impact on cell death sensitivity [28, 29]. On the contrary, healthy liver donor transplant is likely less sensitive to increase in metabolic demand per unit of tissue and cell death sensitivity as compared to liver of a patient with underlying chronic disease or a patient being operated to treat a metastatic tumor. Understanding the combinatorial effect of these two intrinsic parameters, which likely vary from patient-to-patient, on the mode of response to injury can help with better characterization of the perioperative conditions under which liver surgery can lead to a successful recovery or failure.

The determination of threshold of liver failure through a phase portrait approach is analogous to detection of tipping point in a complex dynamical system. Tipping point is a “point of no return” that results in a transition from the state of normal functioning to a catastrophic state [30]. Examples of tipping point are widespread, such as extinction of species in ecological systems, and heavy load on electrical grids or internet, etc. Once such a catastrophic state is attained, the system collapses and there is no going back. In the case of resection, the tipping point corresponds to the threshold of resection beyond which the liver cannot recover and will always progress towards reduced mass and failure. Our analysis suggests that the tipping point after resection is dependent on a combination of the level of metabolic load and the extent of cell death sensitivity [31].

We emphasize that the network modeling approach presented in this study is a post hoc analysis of the dynamics of human liver regeneration. Additional work on identifying patient-specific parameters and developing parameter signatures corresponding to different patient groups based on demographics, disease etiology, etc., so that the dynamic modeling can serve as a pre hoc predictive tool that can aid in clinical decision making. Availability of detailed perioperative clinical information opens new opportunities for developing a categorical (e.g., classification-based) or a quantitative relationship between these physiological parameters (M and β_ap_) and patient-specific clinical parameters [18], aiding generalization of the dynamic modeling approach. For example, if the model prediction suggests a likelihood of liver failure following resection, interventions such as portal vein embolization to induce regeneration and enhance pre-resection liver mass [32], preoperative dietary restriction [33] and nutritional changes [34] to reduce the risk of ischemia-reperfusion injury, as well as preoperative reduction in systemic inflammation [35, 36], so as to modulate the metabolic load and cell death sensitivity parameters and thereby shift the likely response to the region of full recovery of liver mass. In addition, the phase portrait technique can be employed to further aid in predicting the likely safe level of resection.

Thus far, the computational modeling efforts by us and others have considered liver as a uniform tissue in a single lumped compartment [6, 8, 9]. Opportunities exist for computational modeling approaches that explicitly consider multiple lobes of differing size with potentially distinct responses to resection [37]. In such a scenario, the metabolic load and cell death sensitivity parameters may need to be considered as heterogeneous across liver tissue. Non-invasive imaging techniques, which are regularly employed in the clinic to obtain whole organ physiological and functional parameters, can aid in evolving the dynamic models in such potentially fruitful directions.

## Conclusions

In the present study, we have extended and fine-tuned a network model of liver regeneration to predict the dynamics of human liver response to resection. Analysis of the computational model helped us identify two crucial factors associated with the metabolic load and cell death post resection, which can control the dynamics of liver regeneration response. Our simulations indicate that the balance between these two factors is critical to drive the response towards liver recovery or failure. We evaluated the distribution of responses in a cohort of virtual patients, and analyzed the responses using phase plane analysis to identify how the threshold of liver failure varies as a function of the model-predicted critical factors. Our analysis demonstrates a model-based approach to estimate the safe level of resection to increase the likelihood of recovery. These results serve as a basis for future efforts focused on relating the two model-predicted critical factors to patient-specific pre- and perioperative clinical parameters to aid in clinical decision making.

## Methods

### Computational model

We employed the mathematical model of Cook et al. [8] which is an extension of the model proposed by Furchtgott et al. [6]. This model describes the hepatocyte growth after partial hepatectomy. In this model, hepatocytes enter the cell cycle and is assumed to exist in one of the three states quiescent (Q), priming (P) or replicating (R) via a cascade of signals from cytokines and growth factors [38, 39]. We assume the liver to be a lumped system, and consequently, the molecular changes in hepatocytes are considered to be spatially homogeneous throughout the remnant liver mass in the present lumped parameter model.

The dynamic response to partial hepatectomy is modeled as governed by 11 ordinary differential equations. Three of these variables represent the hepatocyte proportions in the three distinct functional states (quiescent, primed, and replicating). Seven of the remaining equations describe the dynamics of molecular factors that take into account the molecular regulation of hepatocytes, e.g., cytokines signals from Kupffer cells for priming of hepatocytes, and growth factor signals from hepatic stellate cells. The last equation accounts for the relative cell mass to incorporate hypertrophy along with hyperplasia in the model. In this work, the model proposed by Cook et al. [8] was modified to account for senescent, non-replicating cells by modifying the metabolic load per unit cell number (M / N). This variable was replaced by metabolic load per unit of replication-competent and senescent cells (M / (N+*ε*)), where N represents cells that can grow via hyperplasia and hypertrophy and *ε* represents cells that do not grow but are still functional. For the simulations in the present study, we have assumed 1% cells as functional but not capable of replication, and so *ε* = 0.01. This modification reduces the numerical error encountered in simulations corresponding to liver failure scenarios. The model equations are given as below:

- Cellular states:

1 $$ \frac{dQ}{dt}=-{k}_{QP}\left(\left[ IE\right]-\left[{IE}_0\right]\right)Q+{k}_{RQ}\left[ ECM\right]R+{k}_{req}{\sigma}_{req}P-{k}_{ap}{\sigma}_{ap}Q $$

2 $$ \frac{dP}{dt}={k}_{QP}\left(\left[ IE\right]-\left[{IE}_0\right]\right)Q-{k}_{PR}\left( GF-\left[{GF}_0\right]\right)P-{k}_{req}{\sigma}_{req}P-{k}_{ap}{\sigma}_{ap}P $$

3 $$ \frac{dR}{dt}={k}_{PR}\left(\left[ GF\right]-\left[{GF}_0\right]\right)P-{k}_{RQ}\left[ ECM\right]R+{k}_{prol}R-{k}_{ap}{\sigma}_{ap}R $$

- Molecular factors:

4 $$ \frac{d\left[ IL6\right]}{dt}={k}_{IL6}\frac{M}{N+\varepsilon }-\frac{V_{JAK}\left[ IL6\right]}{\left[ IL6\right]+{K}_M^{JAK}}-{\kappa}_{IL6}\left[ IL6\right]+{k}_1 $$

5 $$ \frac{d\left[ JAK\right]}{dt}=\frac{V_{JAK}\left[ IL6\right]}{\left[ IL6\right]+{K}_M^{JAK}}-{\kappa}_{JAK}\left[ JAK\right]+{k}_2 $$

6 $$ \frac{d\left[ STAT3\right]}{dt}=\frac{V_{ST3}\left[ JAK\right]{\left[ proSTAT3\right]}^2}{{\left[ proSTAT3\right]}^2+{K}_M^{ST3}\left(1+\left[ SOCS3\right]/{K}_I^{SOCS3}\right)}-\frac{V_{IE}\left[ STAT3\right]}{\left[ STAT3\right]+{K}_M^{IE}}-\frac{V_{SOCS3}\left[ STAT3\right]}{\left[ STAT3\right]+{K}_M^{SOCS3}}-{\kappa}_{ST3}\left[ STAT3\right]+{k}_3 $$

7 $$ \frac{d\left[ SOCS3\right]}{dt}=\frac{V_{SOCS3}\left[ STAT3\right]}{\left[ STAT3\right]+{K}_M^{SOCS3}}-{\kappa}_{SOCS3}\left[ SOCS3\right]+{k}_4 $$

8 $$ \frac{d\left[ IE\right]}{dt}=\frac{V_{IE}\left[ STAT3\right]}{\left[ STAT3\right]+{K}_M^{IE}}-{\kappa}_{IE}\left[ IE\right]+{k}_5 $$

9 $$ \frac{d\left[ GF\right]}{dt}={k}_{GF}\frac{M}{N+\varepsilon }-{k}_{up}\left[ GF\right]\left[ ECM\right]-{\kappa}_{GF}\left[ GF\right]+{k}_7 $$

10 $$ \frac{d\left[ ECM\right]}{dt}=-{k}_{\mathrm{deg}}\left[ IL6\right]\left[ ECM\right]-{\kappa}_{ECM}\left[ ECM\right]+{k}_6 $$

- Relative cell mass:

11 $$ \frac{dG}{dt}={k}_G\left(\frac{M}{N+\varepsilon}\right)-{k}_GM $$

where,

12 $$ \varepsilon =0.01 $$

13 $$ {\sigma}_{ap}=0.5\left(1+\tanh \left(\frac{\theta_{ap}-\left(N+\varepsilon \right)/M}{\beta_{ap}}\right)\right) $$

14 $$ {\sigma}_{req}=0.5\left(1+\tanh \left(\frac{\theta_{req}-\left[ GF\right]}{\beta_{req}}\right)\right) $$

15 $$ N=Q+G\left(P+R\right) $$

16 $$ {k}_1=\frac{V_{JAK}}{1+{K}_M^{JAK}}-{k}_{IL6}\frac{M}{N_{ss}+\varepsilon }+{\kappa}_{IL6} $$

17 $$ {k}_2={\kappa}_{JAK}-\frac{V_{JAK}}{1+{K}_M^{JAK}} $$

18 $$ {k}_3=-\frac{V_{ST3}{\left[ proSTAT3\right]}^2}{{\left[ proSTAT3\right]}^2+{K}_M^{ST3}\left(1+1/{K}_I^{SOCS3}\right)}+\frac{V_{IE}}{1+{K}_M^{IE}}+\frac{V_{SOCS3}}{1+{K}_M^{SOCS3}}+{\kappa}_{ST3} $$

19 $$ {k}_4=-\frac{V_{SOCS3}}{1+{K}_M^{SOCS3}}+{\kappa}_{SOCS3} $$

20 $$ {k}_5=-\frac{V_{IE}}{1+{K}_M^{IE}}+{\kappa}_{IE} $$

21 $$ {k}_6={k}_{\mathrm{deg}}+{\kappa}_{ECM} $$

22 $$ {k}_7=-{k}_{GF}\frac{M}{N_{SS}+\varepsilon }+{k}_{up}+{\kappa}_{GF} $$

23 $$ {N}_{SS}=0.99 $$

Here, the parameters k_1_ … k_7_ are defined such that the rate of change of molecular species over time is set to zero under normal functioning of the liver, i.e. a steady state, prior to resection. These parameters correspond to homeostatic in- and out-fluxes in the liver at steady state, and are not altered during the liver regeneration process. N_SS_ is the steady state of the non-senescent liver mass before resection. N_SS_ and ε together constitute the steady state of the total liver mass prior to surgery.

- Initial conditions:

24 $$ {\displaystyle \begin{array}{l}{Q}_0=\mathrm{remnant}\kern0.34em \mathrm{liver}\kern0.34em \mathrm{fraction};{P}_0=0;{R}_0=0\\ {}\left[ IL{6}_0\right]=1;\left[{JAK}_0\right]=1;\left[ STAT{3}_0\right]=1;\left[ SOCS{3}_0\right]=1;\left[{IE}_0\right]=1;\left[{GF}_0\right]=1;\left[{ECM}_0\right]=1\\ {}{G}_0=1\\ {}{N}_0={Q}_0+{G}_0\left({P}_0+{R}_0\right)={Q}_0\end{array}} $$

### Simulation and parameter optimization

The Matlab code used for model simulation in this study is available as supplemental information in Additional file 8. Simulations were performed in Matlab using *ode15s*. The initial guess values of the parameters for optimization were based on the values given in Cook et al. [8] for human population. The parameters of the model were optimized using the sparse regularization technique of *elastic net*, which is a combination of ridge regression and Lasso [40, 41]. We sampled the parameter space using Sobol sampling [42, 43] with a few parameters varied over a ten-fold range and the remaining parameters varied within a two-fold range around the initial value (Additional file 9: Table S3). The parameter ranges were so chosen to avoid the numerical integration error since the system of equations for the model is stiff. The Matlab code for parameter optimization is available as supplemental information in Additional file 10.

### Metabolic scaling from rat to human based on body mass

For the comparative analysis of parameter tuning we considered two alternative approaches to multivariate optimization. We calculated the metabolic load (M) based on the empirical scaling relationship between M and body mass, as published in Young et al. [21] and Cook et al. [8]. In the case of Young et al. [21], the M value is calculated as:

25 $$ M=23.409\ x\  Body\ {Mass}^{-0.118} $$

In the case of Cook et al. [8], the M value is calculated as:

26 $$ M=47.315\;x\; Body\;{Mass}^{-0.1825} $$

where Body Mass is in grams in Eqs. (25) and (26).

In addition to M, we also modified the relative cell mass growth constant (k_G_) to 6.5675e-4 when simulating the model according to the parameter tuning published by Cook et al. [8] to translate from rat to the human case.

For each patient, the body mass was calculated from the available body mass index (BMI) data from Yamamoto et al. [18]. The BMI values were converted to body mass values based on average height of Japanese adult men (1.72 m) and women (1.58 m) [44], as follows:

27 $$ Body\ Mass=1000\;x\; BMI\;x\;{height}^2 $$

where BMI is in kg/m^2^, Body Mass is in grams and height is in meters.

### Virtual patient cohort generation

In order to identify the critical factors controlling both the mechanism of liver recovery and failure, we generated in silico cohorts of virtual patients [23, 45] starting with specific patient data (ID71) from Yamamoto et al. [18] and introducing wide variation in specific parameters as detailed in the Results. We utilized a Sobol sampling approach that yields a space-filling sample with little bias [43]. The model-predicted critical factors were first varied one at a time and then in combination to analyze their influence on the individual patient liver response to surgical resection. The Matlab code for virtual patient cohort analysis is available as supplemental information in Additional file 11.

### Decision boundary and threshold of failure

The decision boundary demarcating the normal liver growth from the other classes of liver response were drawn using a support vector machine approach with a third order polynomial kernel using *fitcsvm* function in Matlab with BoxConstraint in the range of 1 to 10^4^, for different levels of resection [46]. For each virtual patient, the threshold of failure was calculated by evaluating the response to varying level of resection, ranging from 5 to 90%. The response was considered as liver failure if the liver mass fraction was below 0.1 at the 2 year time point post-surgery. Responses of a virtual cohort of patients were simulated for different levels of resection to identify the threshold of resection beyond which any given virtual patient undergoes liver failure.

### Response mode analysis

The in silico generated regeneration profiles of different virtual patients are classified as normal growth when the liver mass fraction at 2.5 years post-surgical resection is within 0.9–1.1 and as suppressed mode for fraction below 0.9 for recovered patients. Liver failure modes are those where the liver mass fraction is below 0.1 after a 2 year post surgery. The unresponsive mode corresponds to the case where the virtual patient does not show any change in the liver mass fraction after surgery.

### Clinical data set

The data used for the present work has been obtained from Yamamoto et al. [18], which contained information on liver volume post liver resection in 196 patients. We analyzed the data to identify the subset of patients who recovered fully i.e., the patients whose final liver volume was in the range of 90 to 100% of the preoperative liver volume. There were a total of 101 patients whose liver volume recovered fully. These patients were further categorized based on the temporal profile of liver growth. Some patients showed delayed liver growth, while others exhibited suppressed but continuous liver growth.

### Model reproducibility

Simulations presented in the current work were reproduced independently by a laboratory colleague, not associated with the study, who developed new Matlab code based on the model equations and parameter values included in this manuscript. See Additional file 12: Figure S6 for details. The original and reproduced model are provided in the Additional files 1 and 13.

## Additional files

Additional file 1: **Table S1.** Comparison of the metabolic load calculated by the three different approaches of Young et al. [21], Cook et al. [8] and present multivariate optimization approach with the corresponding sum square errors for fit to the liver volume time series data from Yamamoto et al. [18]. (PDF 29 kb)

Additional file 2: **Figure S1.** Comparison of the three alternative approaches of Young et al. [21], Cook et al. [8] and present multivariate optimization for model tuning to fit liver volume time series data corresponding to patient ID74 from Yamamoto et al. [18]. (PDF 235 kb)

Additional file 3: **Figure S2.** Pairwise correlation between optimized model parameters with clinical data of the 27 patients from Yamamoto et al. [18]. (PDF 268 kb)

Additional file 4: **Figure S3.** Correlation between the optimization-based parameters across 27 patients. (PDF 337 kb)

Additional file 5: **Table S2.** Classification of model parameters of Cook et al. [8] model based on their directional influence on liver response. (PDF 161 kb)

Additional file 6: **Figure S4.** Comparison of the impact of intrinsic perioperative factors, metabolic load (M) and cell death sensitivity (β_ap_) between rat and human liver regeneration scenarios. (PDF 478 kb)

Additional file 7: **Figure S5.** Determining the safe level of resection based on cell death sensitivity (β_ap_), an intrinsic perioperative factor. (PDF 392 kb)

Additional file 8: Matlab code to generate the regeneration profiles and phase plane portrait for predicting the threshold of failure. (M 11 kb)

Additional file 9: **Table S3.** Description of model parameters with their bounds considered in the optimization. (PDF 174 kb)

Additional file 10: Matlab code to optimize the model parameters using elastic net technique. (M 10 kb)

Additional file 11: Matlab code for virtual patient cohort analysis. (M 11 kb)

Additional file 12: **Figure S6.** Model reproducibility. Using the model equations and parameters given in the main text. (PDF 484 kb)

Additional file 13: Matlab code used to reproduce the results of the original model implementation. (M 19 kb)
