# Supplementary material for: Model-based virtual patient analysis of human liver regeneration predicts critical perioperative factors controlling the dynamic mode of response to resection
Source: BMC Syst Biol. 2019 Jan 16;13:9. doi: 10.1186/s12918-019-0678-y (PMC6335689; doi:10.1186/s12918-019-0678-y)
Supplement: Supplementary file 1 — Table S1. Comparison of the metabolic load calculated by the three different approaches of Young et al. [21], Cook et al. [8] and present multivariate optimization approach with the corresponding sum square errors for fit to the liver volume time series data from Yamamoto et al. [18]. (PDF 29 kb) [file 12918_2019_678_MOESM1_ESM.pdf]

Table S1. Comparison of the metabolic load calculated by the three different approaches of Young et al. [21], Cook et al. [8] and present multivariate optimization approach with the corresponding sum square errors for fit to the liver volume time series data from Yamamoto et al. [18].

Patient ID	Young et al.		Cook et al.		Multivariate Optimization	
	M	SSE	M	SSE	M	SSE
4	6.13614	5.986E-02	5.96574	5.830E-02	5.85537	4.652E-02
24	6.28769	2.465E-02	6.19516	2.512E-02	5.81011	1.505E-02
30	6.35691	1.391E-02	6.30095	1.272E-02	5.88455	5.419E-03
31	6.20712	1.057E-01	6.07281	1.057E-01	5.76161	3.928E-02
44	6.36192	1.503E-02	6.30863	1.666E-02	5.78737	8.227E-03
46	6.48053	8.481E-03	6.49147	7.510E-03	5.81837	3.672E-03
71	6.36695	6.429E-02	6.31634	6.178E-02	5.27725	1.662E-03
74	6.20060	1.059E-02	6.06294	1.017E-02	5.85572	8.949E-03
90	6.43058	7.067E-04	6.41424	7.617E-04	5.85930	1.498E-03
93	6.48461	1.609E-02	6.49778	1.360E-02	5.96303	7.922E-04
94	6.41900	2.521E-02	6.39639	2.267E-02	5.82839	2.045E-03
97	6.54534	1.723E-02	6.59214	1.475E-02	5.82589	4.779E-03
104	6.51549	1.441E-03	6.54570	1.468E-03	5.83453	6.000E-04
115	6.47193	6.363E-02	6.47815	6.082E-02	5.84523	6.226E-02
125	6.45081	7.149E-02	6.44548	7.057E-02	5.87353	6.887E-02
133	6.29743	1.790E-02	6.21000	1.655E-02	5.88404	5.952E-03
147	6.30260	1.486E-02	6.21789	1.394E-02	5.82748	8.328E-03
153	6.21445	2.895E-01	6.08390	2.848E-01	5.74923	1.440E-01
156	6.30296	5.525E-03	6.21844	6.045E-03	5.99036	3.134E-03
159	6.18711	6.462E-02	6.04255	6.235E-02	5.88752	4.947E-02
160	6.43077	8.404E-02	6.41453	7.915E-02	5.88308	6.245E-03
176	6.51591	2.505E-02	6.54635	2.457E-02	5.84513	2.208E-02
177	6.46901	4.284E-02	6.47362	3.906E-02	5.87770	1.713E-02
182	6.22332	5.839E-03	6.09733	5.939E-03	5.81962	3.596E-03
186	6.25998	3.077E-02	6.15298	2.985E-02	5.85235	1.986E-02
192	6.33958	3.813E-02	6.27440	3.462E-02	6.10168	8.002E-03
196	6.30296	1.826E-02	6.21844	1.890E-02	6.10168	1.334E-02
