# Supplementary material for: Model-based virtual patient analysis of human liver regeneration predicts critical perioperative factors controlling the dynamic mode of response to resection
Source: BMC Syst Biol. 2019 Jan 16;13:9. doi: 10.1186/s12918-019-0678-y (PMC6335689; doi:10.1186/s12918-019-0678-y)
Supplement: Supplementary file 2 — Figure S1. Comparison of the three alternative approaches of Young et al. [21], Cook et al. [8] and present multivariate optimization for model tuning to fit liver volume time series data corresponding to patient ID74 from Yamamoto et al. [18]. (PDF 235 kb) [file 12918_2019_678_MOESM2_ESM.pdf]

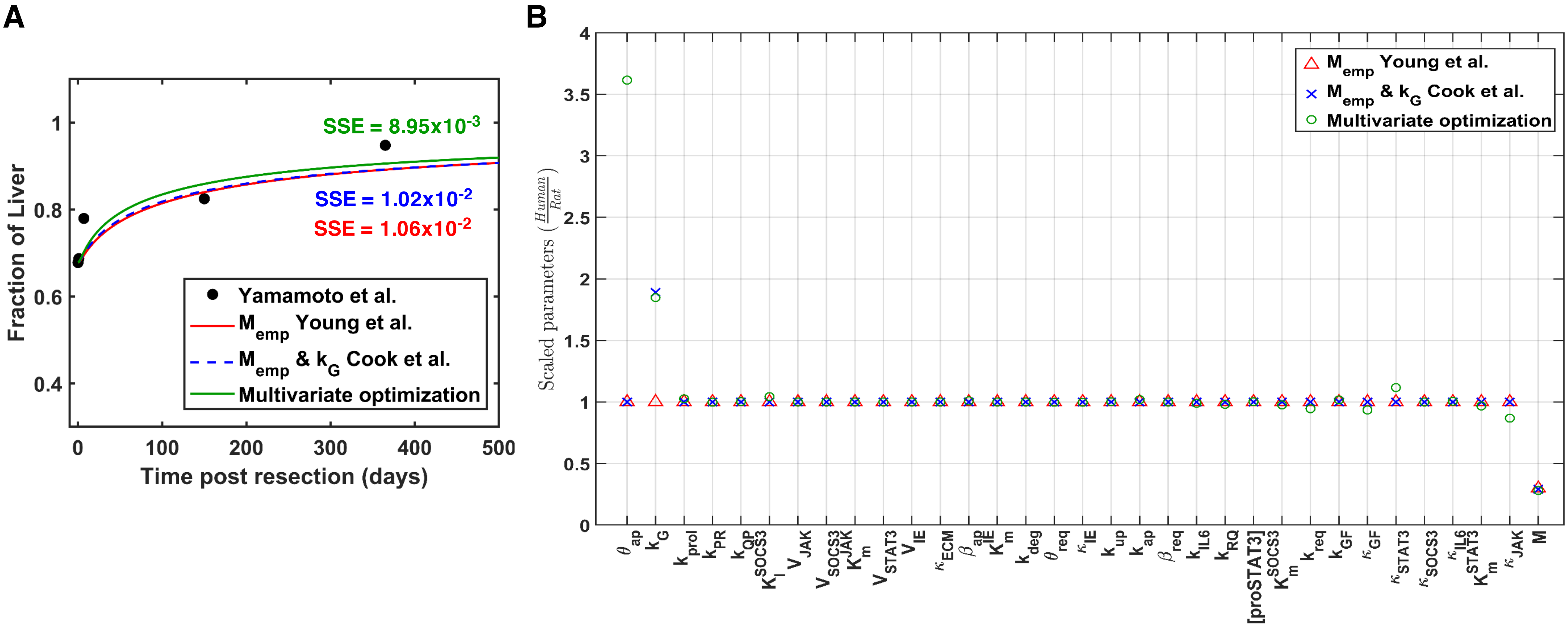

Figure S1: Comparison of the three alternative approaches of Young et al. [21], Cook et al. [8] and present multivariate optimization for model tuning to fit liver volume time series data corresponding to patient ID74 from Yamamoto et al. [18]. (A) Comparison of the regeneration profiles shows that the Young et al. [21] approach of empirically scaling only the metabolic load captures the dynamics of liver regeneration reasonably for this patient while the other two approaches improved the fit marginally. (B) Relative variation of all the 33 model parameters for the three approaches scaled by Cook et al. [8] parameters for rat. Our results suggest that while a subset of patients need multivariate scaling of model parameters, others show a good fit with scaling the metabolic load alone.
