# Supplementary material for: Model-based virtual patient analysis of human liver regeneration predicts critical perioperative factors controlling the dynamic mode of response to resection
Source: BMC Syst Biol. 2019 Jan 16;13:9. doi: 10.1186/s12918-019-0678-y (PMC6335689; doi:10.1186/s12918-019-0678-y)
Supplement: Supplementary file 3 — Figure S2. Pairwise correlation between optimized model parameters with clinical data of the 27 patients from Yamamoto et al. [18]. (PDF 268 kb) [file 12918_2019_678_MOESM3_ESM.pdf]

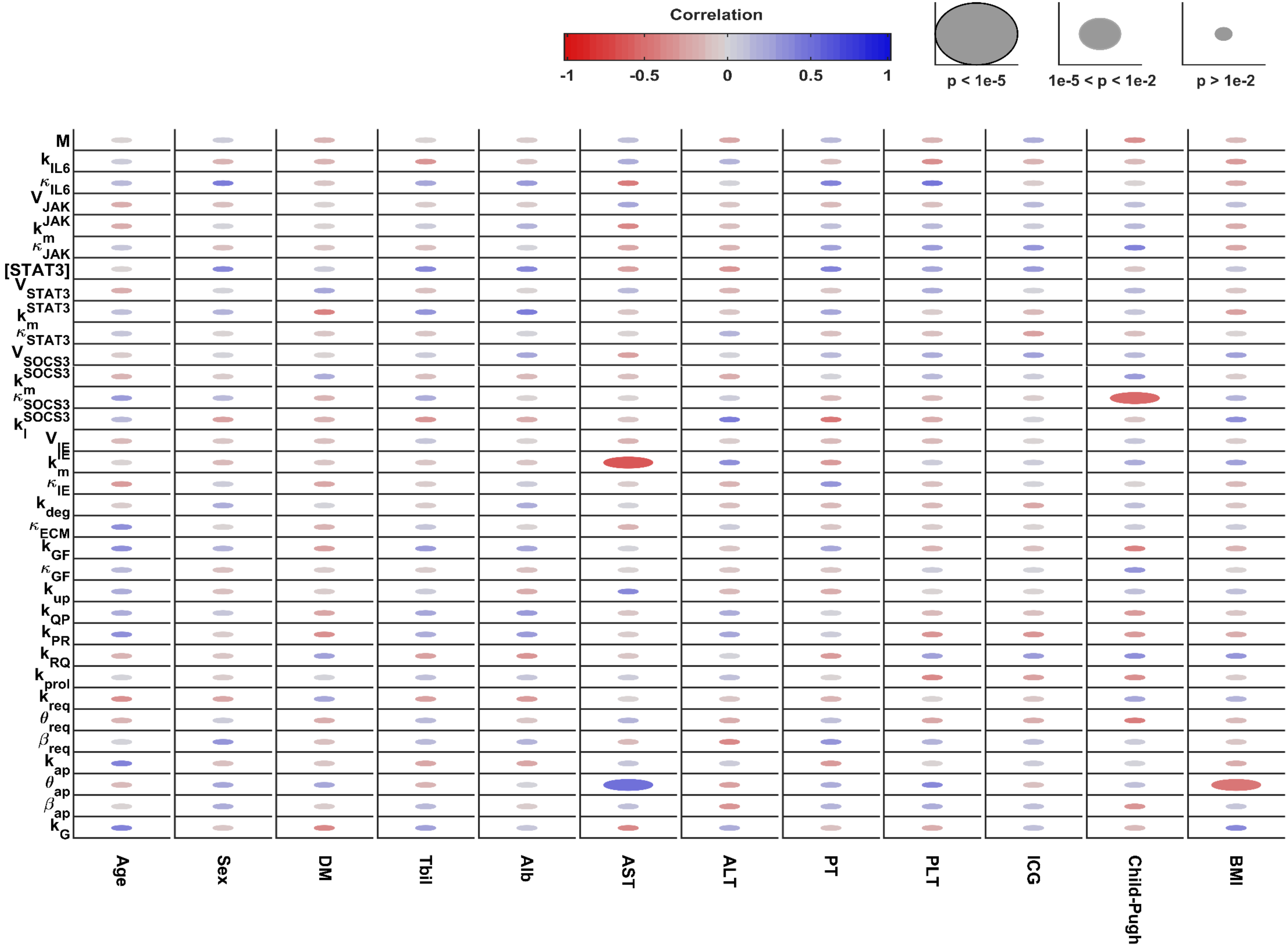

Figure S2: Pairwise correlation between optimized model parameters with clinical data of the 27 patients from Yamamoto et al. [18]. There is no significant pairwise correlation between parameters and the available clinical information (Bonferroni corrected p-value < 1e-5).
