# Supplementary figures and images for: Model-based virtual patient analysis of human liver regeneration predicts critical perioperative factors controlling the dynamic mode of response to resection
Source: BMC Syst Biol. 2019 Jan 16;13:9. doi: 10.1186/s12918-019-0678-y (PMC6335689; doi:10.1186/s12918-019-0678-y)

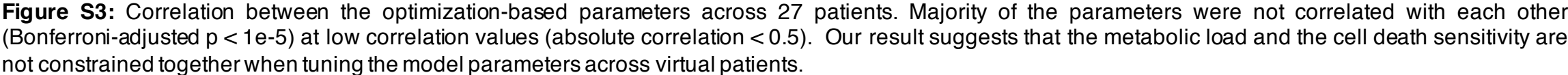

Supplement: Supplementary file 4 — Figure S3. Correlation between the optimization-based parameters across 27 patients. (PDF 337 kb) [file 12918_2019_678_MOESM4_ESM.pdf]
