# Supplementary material for: Model-based virtual patient analysis of human liver regeneration predicts critical perioperative factors controlling the dynamic mode of response to resection
Source: BMC Syst Biol. 2019 Jan 16;13:9. doi: 10.1186/s12918-019-0678-y (PMC6335689; doi:10.1186/s12918-019-0678-y)
Supplement: Supplementary file 5 — Table S2. Classification of model parameters of Cook et al. [8] model based on their directional influence on liver response. (PDF 161 kb) [file 12918_2019_678_MOESM5_ESM.pdf]

Table S2: Model parameters categorized according to the effect on the liver response outcome in the Cook et al. [8] model. Due to numerical difficulties in integrating the Cook et al. [8] model for certain parameter values, the effects of variation in the parameters M, k_{IL6}, k_{prol}, θ_{ap}, β_{ap} were evaluated in a more restricted range than the present model.

<table>
<tr><td colspan="3">Only recovery</td><td colspan="2">Both recovery and failure</td></tr>
<tr><td colspan="2">Sensitive</td><td>Insensitive</td><td>Sensitive</td><td>Insensitive</td></tr>
<tr><td>Improves recovery</td><td>Decelerate recovery</td><td></td><td></td><td></td></tr>
<tr><td>$k_{IL6}, [prosSTAT3], \kappa_{SOCS3}, K_I^{SOCS3}, V_{IE}, k_{\deg}, k_{GF}, k_{QP}, k_{PR}, k_{prol}, \beta_{req}, k_G$</td><td>$\kappa_{IL6}, K_M^{JAK}, \kappa_{JAK}, K_M^{ST3}, V_{SOCS3}, K_M^{SOCS3}, K_M^{IE}, \kappa_{IE}, \kappa_{ECM}, \kappa_{GF}, k_{up}, k_{RQ}, k_{req}$</td><td>$V_{JAK}, V_{ST3}, \kappa_{ST3}, \theta_{req}, k_{ap}$</td><td>$M, \beta_{ap}$</td><td>$\theta_{ap}$</td></tr>
</table>
