# Supplementary material for: Model-based virtual patient analysis of human liver regeneration predicts critical perioperative factors controlling the dynamic mode of response to resection
Source: BMC Syst Biol. 2019 Jan 16;13:9. doi: 10.1186/s12918-019-0678-y (PMC6335689; doi:10.1186/s12918-019-0678-y)
Supplement: Supplementary file 6 — Figure S4. Comparison of the impact of intrinsic perioperative factors, metabolic load (M) and cell death sensitivity (βap) between rat and human liver regeneration scenarios. (PDF 478 kb) [file 12918_2019_678_MOESM6_ESM.pdf]

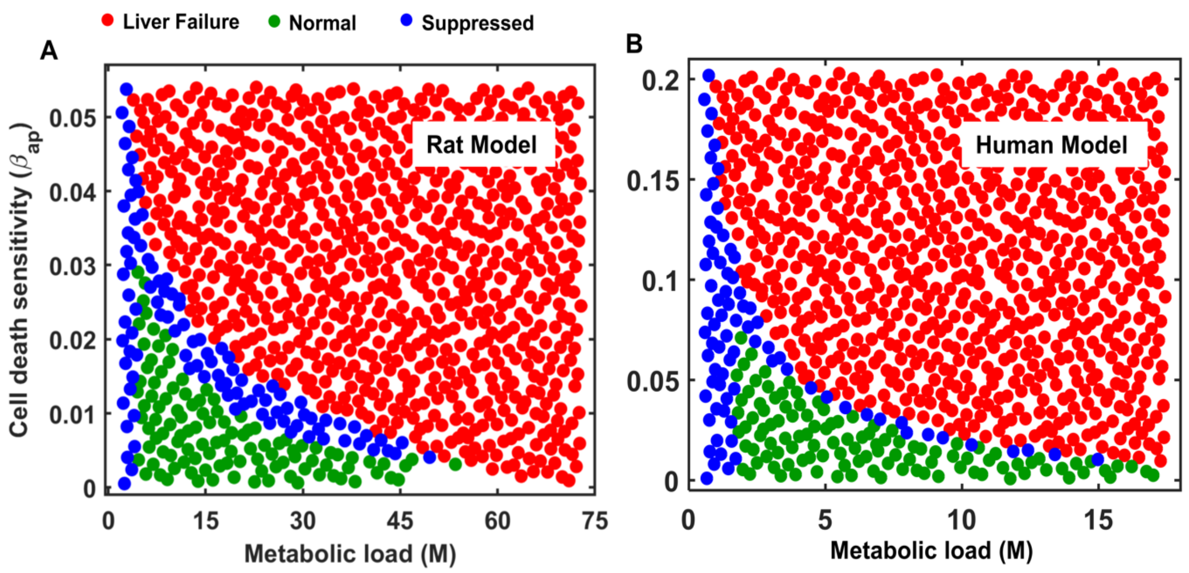

Figure S4: Comparison of the impact of intrinsic perioperative factors, metabolic load (M) and cell death sensitivity (β_{ap}) in rat and human for 33.3% resection, using the present model (A) with rat specific parameters from Cook et al. [8] and (B) with human specific parameters from Table 1. The two perioperative factors showed similar classification in the parameter space in both rat as well as human. The parameter subspace corresponding to suppressed mode enveloped the zone of normal recovery, and acts as a threshold beyond which the parameter changes may lead to liver failure in both rat and human liver regeneration.
