# Supplementary material for: Model-based virtual patient analysis of human liver regeneration predicts critical perioperative factors controlling the dynamic mode of response to resection
Source: BMC Syst Biol. 2019 Jan 16;13:9. doi: 10.1186/s12918-019-0678-y (PMC6335689; doi:10.1186/s12918-019-0678-y)
Supplement: Supplementary file 7 — Figure S5. Determining the safe level of resection based on cell death sensitivity (βap), an intrinsic perioperative factor. (PDF 392 kb) [file 12918_2019_678_MOESM7_ESM.pdf]

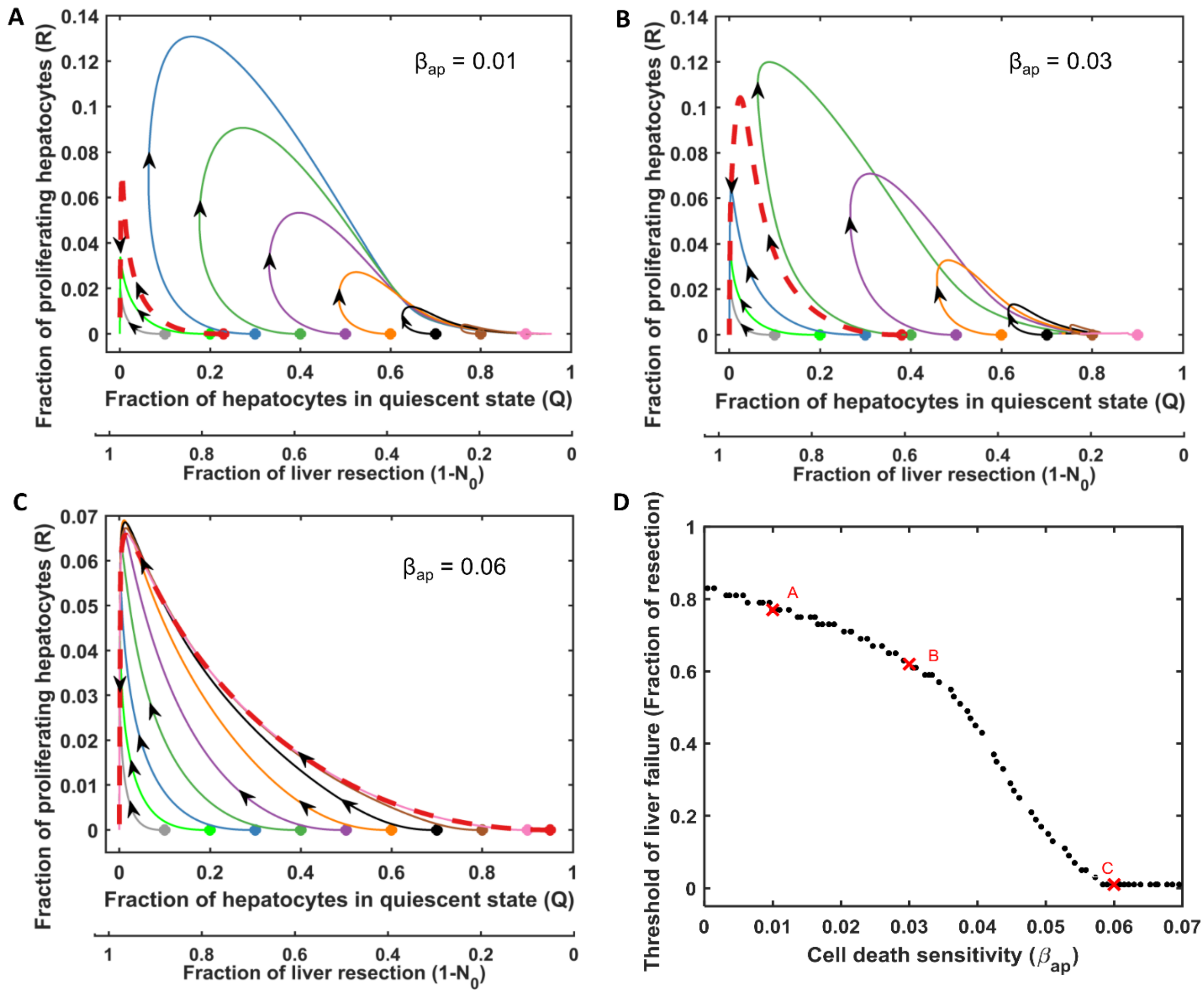

Figure S5: Determining the safe level of resection based on cell death sensitivity (β_{ap}), an intrinsic perioperative factor. (A-C) Phase portrait of quiescent and replicating cell fractions for varying levels of cell death sensitivity parameter (β_{ap}). All other parameters were set to the optimal levels given in Table 1 of the manuscript. The filled circle markers in A-C represent different levels of resection. The red dashed curves represent trajectories for the critical level of resection at and above which failure occurs. (A) β_{ap} =0.01, yields a threshold of liver failure at 77% resection. (B) β_{ap} =0.03, yields a threshold of liver failure at 62% resection. (C) β_{ap} = 0.06, for which all levels of resection lead to failure. Therefore, if cell death sensitivity of a virtual patient is high, the attractor of liver recovery vanishes leading to only one attractor of liver failure. As a consequence, even the smallest level of resection in such patients is likely to result in liver failure, suggesting that surgical resection may not be a viable option. (D) Influence of cell death sensitivity parameter β_{ap} on the threshold of liver failure, which monotonically decreased with increasing cell death sensitivity. Red cross markers in panel D represent the threshold of failure for the corresponding phase planes A-C. Our results suggest that increasing β_{ap} results in the shrinkage of the attractor for liver recovery with a simultaneous increase in the attractor region for liver failure.
