# Supplementary material for: Model-based virtual patient analysis of human liver regeneration predicts critical perioperative factors controlling the dynamic mode of response to resection
Source: BMC Syst Biol. 2019 Jan 16;13:9. doi: 10.1186/s12918-019-0678-y (PMC6335689; doi:10.1186/s12918-019-0678-y)
Supplement: Supplementary file 9 — Table S3. Description of model parameters with their bounds considered in the optimization. (PDF 174 kb) [file 12918_2019_678_MOESM9_ESM.pdf]

Table S3: Biological description of each of the model parameters with their upper and the lower bounds used for optimization via elastic net with Ridge and Lasso weightage of 10^{-3} :

Parameter	Lower bound	Upper bound	Description
M	0.580	58	Mechanical stress due to nutrient and detoxification demand
k_{IL6}	0.150	15	Production rate constant of IL6 from non-parenchymal cells
κ_{IL6}	0.090	9	Degradation rate of IL6
V_{JAK}	2000	200000	Maximum rate of JAK activation
K_M^{JAK}	1000	100000	Michaelis-Menten concentration of JAK
κ_{JAK}	0.040	4	Rate of degradation of JAK
[proSTAT3]	0.010	20	Relative concentration of monomeric STAT3
V_{ST3}	75.0	7500	Maximum rate of STAT3 phosphorylation
K_M^{ST3}	0.040	40	Michaelis-Menten concentration of proSTAT3
κ_{ST3}	0.050	1	Rate of dephosphorylation of proSTAT3
V_{SOCS3}	14000	34000	Maximum rate of SOCS3 activation
K_M^{SOCS3}	0.000	0.001	Michaelis-Menten concentration of SOCS3
κ_{SOCS3}	0.100	0.700	Degradation rate of SOCS3
K_I^{SOCS3}	0.005	0.025	Inhibition constant of SOCS3
V_{IE}	150	350	Maximum rate of IE gene activation
K_M^{IE}	15	21	Concentration of IE gene, when it's activation rate is half the maximum rate
κ_{IE}	3	7	Degradation rate of IE gene
k_{deg}	5.000	9	Degradation rate of ECM by MMPs
κ_{ECM}	30.000	36	Degradation rate of ECM

k_{GF}	0.050	0.20	Rate constant for production of growth factor from non-parenchymal cells
κ_{GF}	0.100	0.35	Degradation rate constant of growth factor
k_{up}	0.040	0.08	Growth factor binding rate to ECM
k_{QP}	0.005	0.009	Transition rate of hepatocytes from quiescence to primed state
k_{PR}	0.003	0.006	Transition rate of hepatocytes from primed to replicating state
k_{RQ}	0.040	0.065	Transition rate of hepatocytes from replicating to quiescence state
k_{prol}	0.010	0.03	Proliferation rate of hepatocytes
k_{req}	0.050	0.15	Transition rate of hepatocytes from primed to quiescent state
θ_{req}	6.000	10	Requiescence parameter in the sigmoidal function (σ_{req}) defining the threshold of hepatocytes transition from primed to quiescent state
β_{req}	2.000	4	Requiescence parameter in the sigmoidal function (σ_{req}) defining the threshold of hepatocytes transition from primed to quiescent state
k_{ap}	0.050	0.150	Cell death rate constant of damaged hepatocytes
θ_{ap}	0.030	0.044	Cell death sensitivity parameter of the sigmoidal function (σ_{ap})
β_{ap}	0.003	0.006	Cell death sensitivity parameter of the sigmoidal function (σ_{ap})
k_G	0.0003	0.0007	Relative cell mass growth rate constant
