# Supplementary material for: Model-based virtual patient analysis of human liver regeneration predicts critical perioperative factors controlling the dynamic mode of response to resection
Source: BMC Syst Biol. 2019 Jan 16;13:9. doi: 10.1186/s12918-019-0678-y (PMC6335689; doi:10.1186/s12918-019-0678-y)
Supplement: Supplementary file 12 — Figure S6. Model reproducibility. Using the model equations and parameters given in the main text. (PDF 484 kb) [file 12918_2019_678_MOESM12_ESM.pdf]

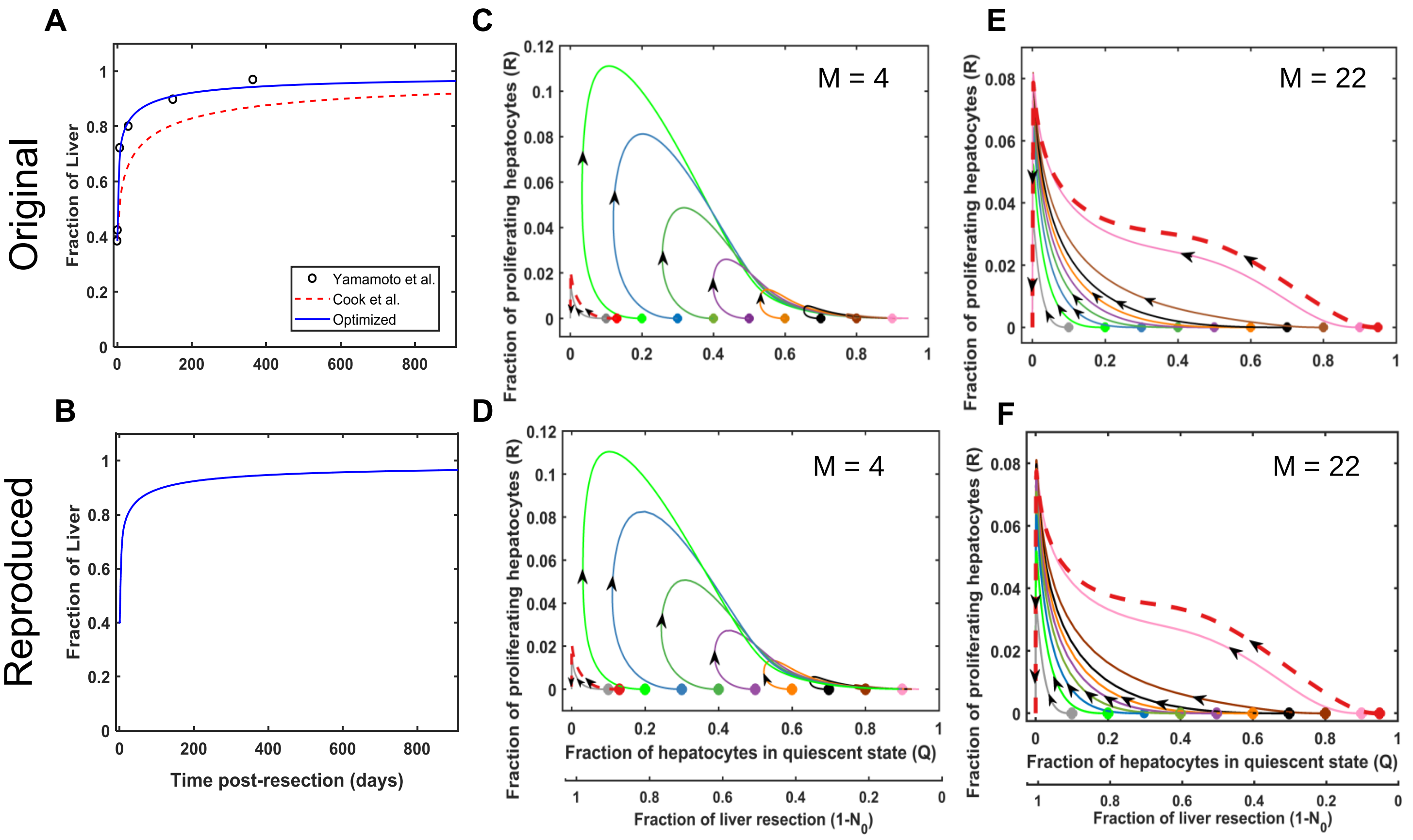

Figure S6: Model reproducibility. The simulation results presented in this work were independently reproduced using the model equations and the parameters given in the main text. **(A-B)**: Original and reproduced regeneration profiles are shown in panels (A) and (B), respectively. Optimized parameter values given in Table 1 of the main text were used to reproduce the regeneration profile. **(C-F)** show that the reproduced phase plane yields the same behavior as the original phase plane for the virtual patients with optimized parameters given in Table 1 of the main text and the metabolic load as mentioned in the figure.
